# Protocol for differential multi-omic analyses of distinct cell types in the mouse cerebral cortex

**DOI:** 10.1016/j.xpro.2023.102793

**Published:** 2023-12-28

**Authors:** Durga Praveen Meka, Melanie Richter, Tabitha Rücker, Hannah Voss, Anne Rissiek, Christoph Krisp, Nisha Hemandhar Kumar, Birgit Schwanke, Eugenio F. Fornasiero, Hartmut Schlüter, Froylan Calderon de Anda

**Affiliations:** 1 RG Neuronal Development, Center for Molecular Neurobiology, University Medical Center Hamburg-Eppendorf, 20251 Hamburg, Germany; 2 Institute for Clinical Chemistry and Laboratory Medicine, Mass Spectrometric Proteomics Group, Campus Forschung, University Medical Center Hamburg-Eppendorf, 20246 Hamburg, Germany; 3 Cytometry und Cell Sorting Core Unit, Department of Stem Cell Transplantation, University Medical Centre Hamburg-Eppendorf, Hamburg, Germany; 4 Department of Neuro- and Sensory Physiology, University Medical Center Göttingen, 37073 Göttingen, Germany; 5 Department of Life Sciences, University of Trieste, 34127 Trieste, Italy; 6 Diagnostic Center, Section Mass Spectrometric Proteomics Group, Campus Forschung, University Medical Center Hamburg-Eppendorf, 20246 Hamburg, Germany

**Keywords:** Bioinformatics, Genomics, RNA-seq, Neuroscience, Proteomics

## Abstract

Here, we present a protocol for differential multi-omic analyses of distinct cell types in the developing mouse cerebral cortex. We describe steps for *in utero* electroporation, subsequent flow-cytometry-based isolation of developing mouse cortical cells, bulk RNA sequencing or quantitative liquid chromatography-tandem mass spectrometry, and bioinformatic analyses. This protocol can be applied to compare the proteomes and transcriptomes of developing mouse cortical cell populations after various manipulations (e.g., epigenetic).

For complete details on the use and execution of this protocol, please refer to Meka et al. (2022).^1^

## Highlights

• Labeling and isolation of mouse cortical cells by IUE and FACS, respectively
• Simultaneous analysis of the cell population across different modalities in bulk
• Comparison of cells from mouse embryos with genetic or pharmacological modification
• Quantitative comparison of proteomes using LC-MS/MS and bioinformatics

## Abstract (editor highlights)

Publisher’s note: Undertaking any experimental protocol requires adherence to local institutional guidelines for laboratory safety and ethics.

## Graphical abstract

**Figure:**
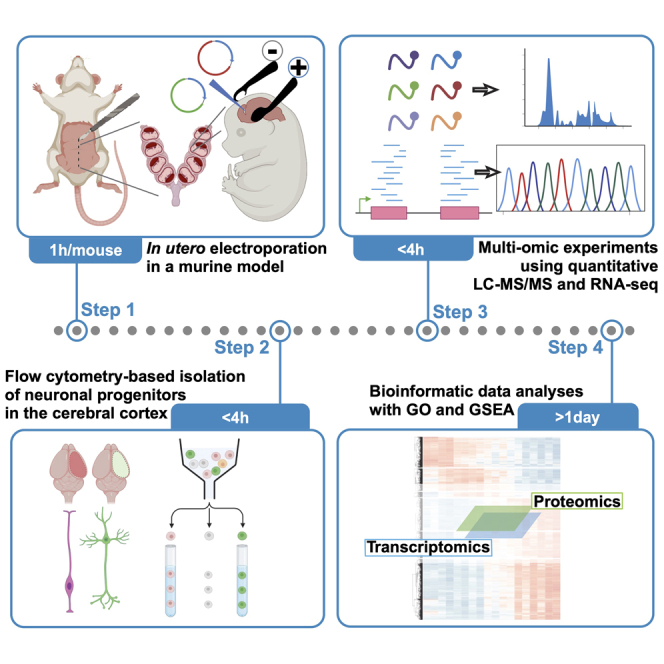

## Before you begin

We recently published the protocol of Meka et al., 2022^1^ that employs a liquid chromatography-tandem mass spectrometry (LC-MS/MS)-based approach to study the effect of acute (shRNA-mediated) downregulation of centrosomal protein 120 (CEP120) on the proteome of cortical neurons in developing mouse brains. CEP120 crucially regulates microtubule stability, which was altered after intervention with shRNA. We applied this protocol to analyze changes in protein expression involved in axonal formation and elongation, and polarized cell growth; all biological processes that occur during the axonal extension of a neuron.^1^ This indicates that the approach described can successfully analyze distally localized and less frequently expressed proteins, even in migrating neurons.

**Figure 1:**
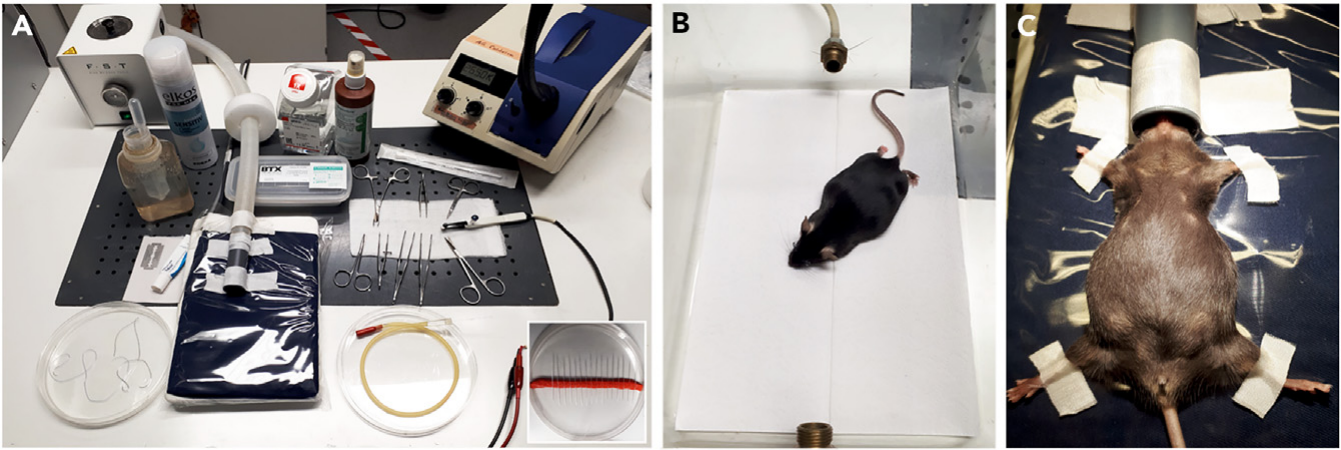
*In utero* electroporation of an anaesthetized mouse (A) Workspace setup, cold source lamps, and surgery tools for *in utero* electroporation. Insert: Pulled borosilicate micropipettes stored on play dough in a 15-cm cell culture plate. (B) Right before surgery, the mouse is anesthetized in an isoflurane chamber and the eyes are covered with Vidisic gel to prevent drying out of eyes. (C) Fixation of the mouse with lab tape on its back on a heating pad with an isoflurane vaporizer nozzle fitting on the nose of the mouse.

**Figure 2:**
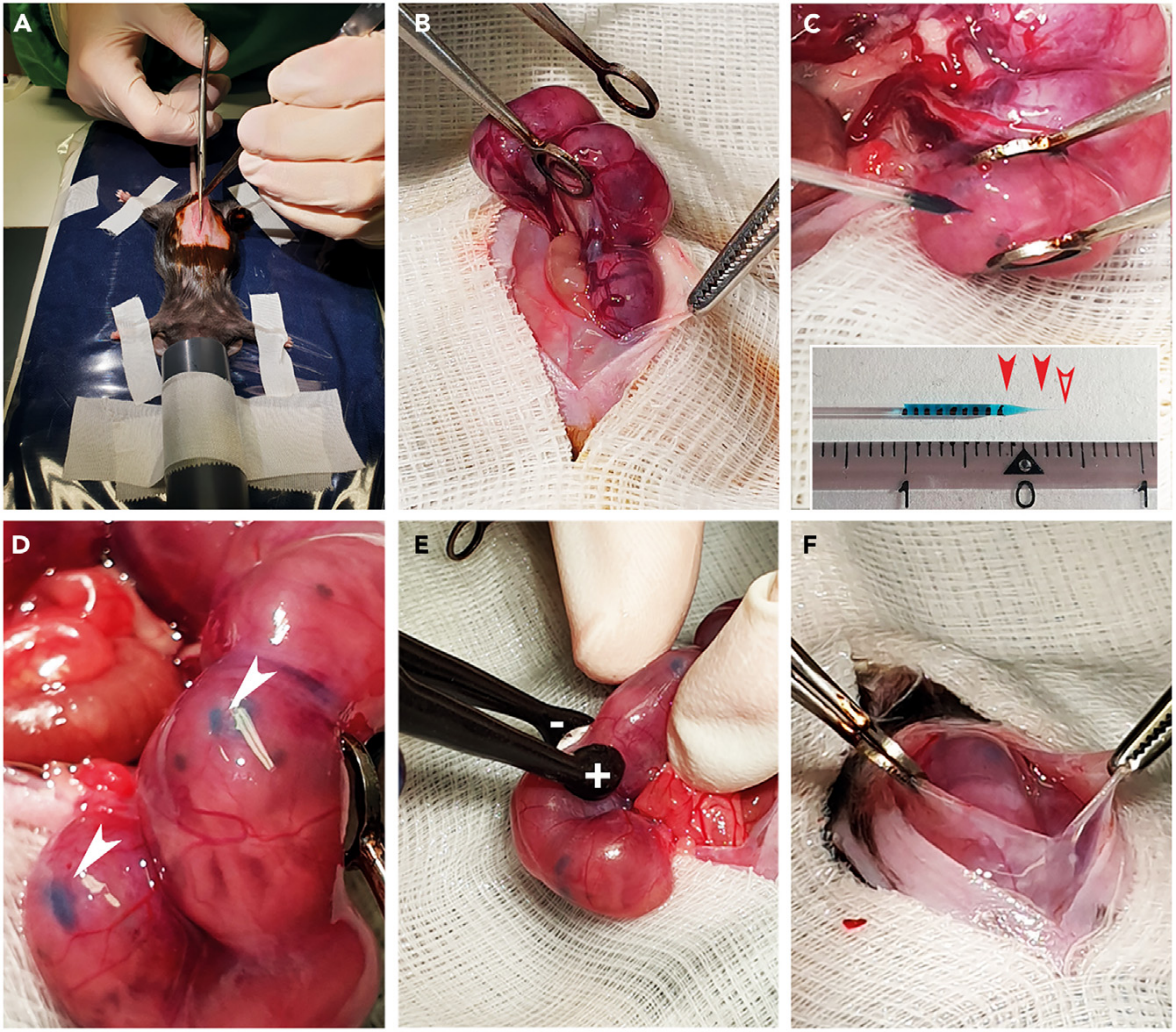
Laparotomy and *in utero* electroporation of an anesthetized mouse (A) After disinfection, the abdomen is shaved manually, and the skin and abdominal cavity are opened longitudinally along the midline using fine scissors. (B) Taking out the uterus horn with ring forceps. (C) Injection of shRNA or DNA plasmid/Fast Green solution into one ventricle. Insert: Pulled borosilicate pipette with 1-mm marks filled with diluted Fast Green solution. Filled arrowheads indicate the tapered shoulder and an empty arrowhead marks the ∼2-mm tip after the cut-off. (D) Ventricles appear in a crescent-shaped form after successful injection (white arrowheads). (E) Delivering electric pulses across the brain of the embryo using a platinum plate tweezers-type electrode to target the lateral ventricles. + indicates the anode, - indicates the cathode. (F) Placing the embryos back into the abdominal cavity and filling up the cavity with warm saline. Pulling up both sides of the incision site to slide embryos back to their original position.

**Figure 3:**
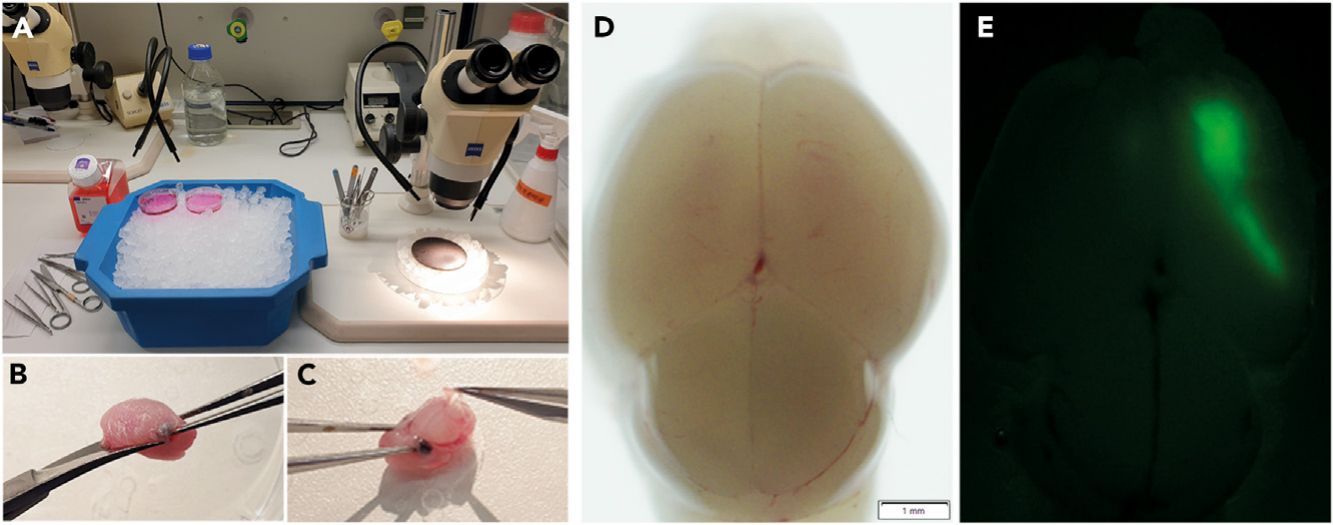
Cortical cell preparation (A) Workspace setup for cortical neuron preparation. (B) Incision to remove the skin from the head. (C) Removal of skull bone and meninges. (D and E): Isolated mouse brain seen under the stereomicroscope: Bright field image shown in (D) and cortical region transfected with an eGFP-expressing plasmid under UV light seen in (E). Scale bar: 1 mm.

**Figure 4:**
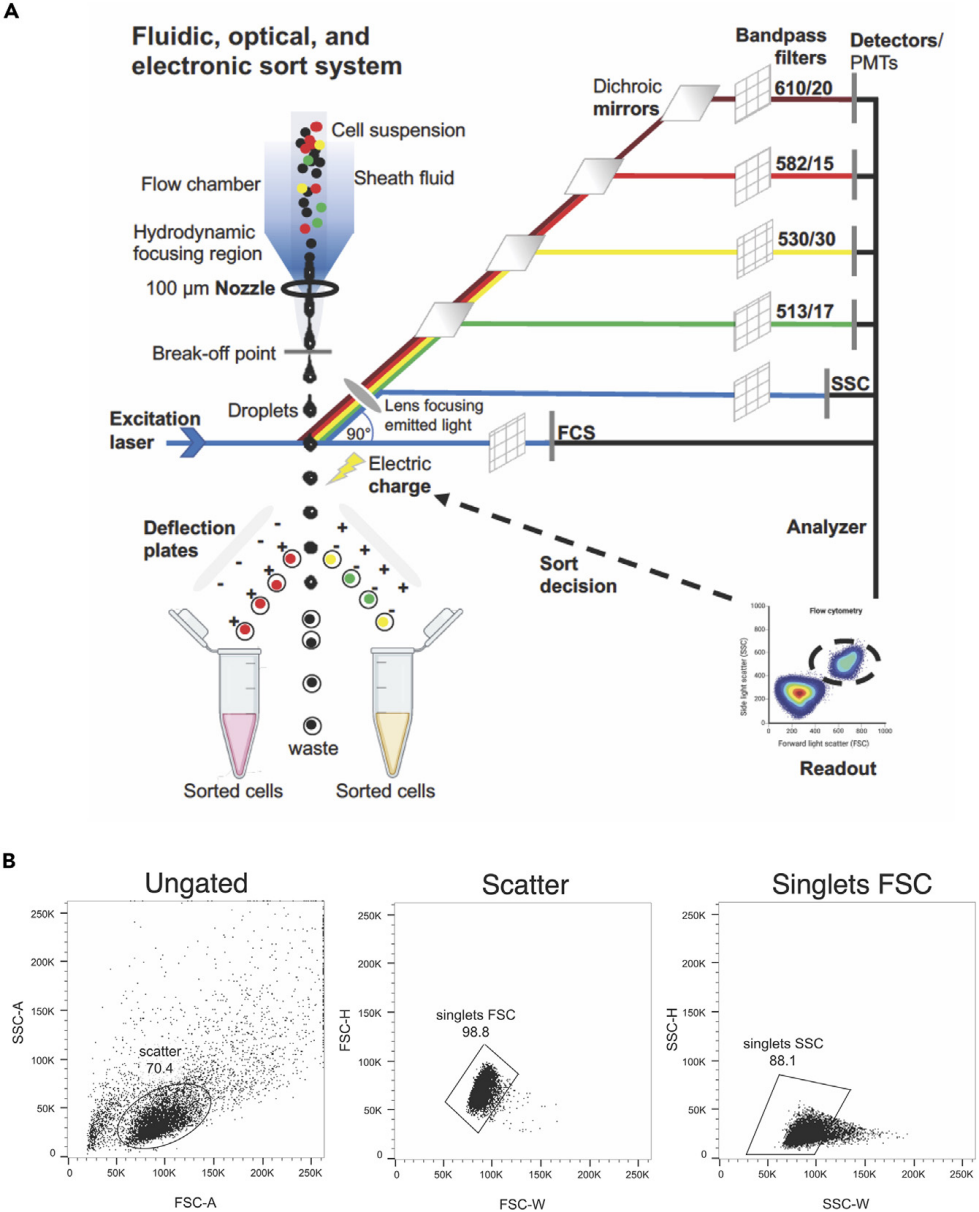
Principle of flow cytometry for sorting early neurons (A) The cell suspension containing transfected and non-transfected cells passes through a 100 μm nozzle and undergoes hydrodynamic focusing within the flow cell due to pressure differences between the sheath fluid and the cell suspension. After separating the stream into single droplets containing one or more cells, the excitation laser interrogates each droplet. Depending on the size (forward scatter, FSC), granularity (side scatter, SSC), and wavelength of the emitted light, a specific sorting decision is formulated by the user. Mirrors deflect the incoming laser beam and filters pass a specific wavelength range to the respective detectors. The fluorophores used here required bandpass filters of 610/20 (dsRed2), 582/15 (tDimer), 530/30 (Venus), and 513/17 (eGFP). The resulting conversion of light to an electrical charge determines the deflection of cells into their respective sorting tubes. Non-transfected cells are not charged and go to the waste container. PTM = Photomultiplier tube. (B) Each sort decision begins with identifying the cell population of interest by plotting SSC-area (SSC-A) against FSC-A to compare the size and granularity of the scatter. Second, duplicates in this scatter were excluded by matching the width (W) against the height (H) of the FSC. These gated FSC singlets served as input for the detected cells in the SSC-H versus SSC-W plot. *The resulting****SSC singlet cell population****served as the final input for differential fluorescence detection every time the respective transfected cells were sorted (see*Figures 5B and 6B*) and was employed for sorting the constructs used here.*

**Figure 5:**
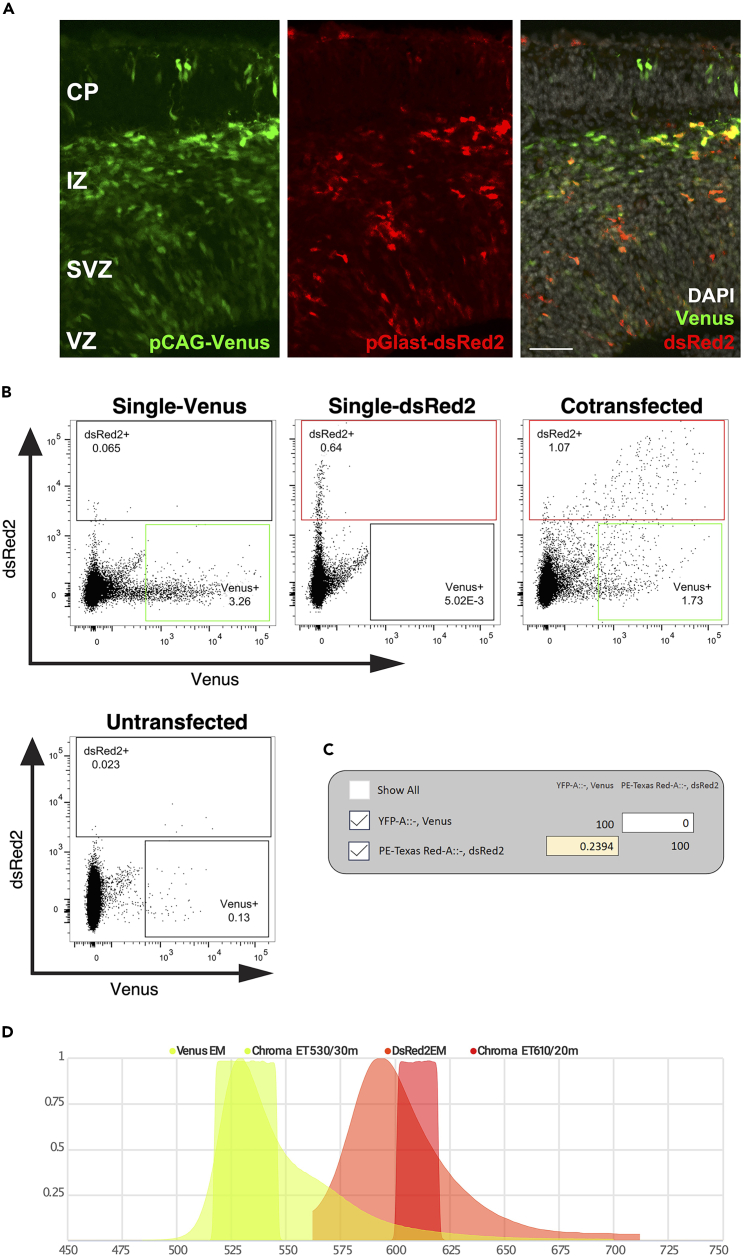
Gating strategy for separating a cell population into subpopulations by flow cytometry based on the transfection of the construct pGlast-dsRed2/pCAG-Venus (A) Coronal sections of the murine somatosensory cortex of embryonal age E14 after IUE at E12 showing fluorescent protein expression in the target cell population: Venus expression driven by the ubiquitous promoter pCAG (**left**), dsRed2 expression driven by apical radial glia cell specific promoter Glast (**middle**), and merged (**right**). CP: cortical plate, IZ: intermediate zone, SVZ: subventricular zone, VZ: ventricular zone. Scale bar: 50 μm. (**B) Singlets SSC populations** of both fluorescence minus one (FMO, single transfected) controls, untransfected, and cotransfected samples with the construct pCAG-Venus/pGlast-dsRed2. The FMO control of the single Venus transfection and the FMO control of the single dsRed2 transfection, together with the untransfected control, were used to calculate the compensation. Compensation with the FMO signals brighter than the signal in the cotransfected sample ensured correct differential fluorescence detection in the cotransfected sample. This compensation and gate setting served as template every time the cotransfected samples were sorted. **Note**: Based on our experimental question, dsRed2+ cells may also have Venus fluorescence (double positive). This experiment was performed on 20,000 cells of E14 embryonic cortices. At this stage, an additional low false positive signal was visible already in the untransfected condition, which was excluded for sorting. (C) Computational compensation matrix (“Compensation Wizard” of BD FACS Diva software; recreated with FlowJo) defined values for compensation of spillover into the other bandpass filter. (D) The bandpass filter (BP) 530/30 covered the emission peak and the spread in the case of FP Venus well, while the BP filter (610/20) covered the emission spectrum of dsRed2 best. The plot was created with FPbase.^17^

**Figure 6:**
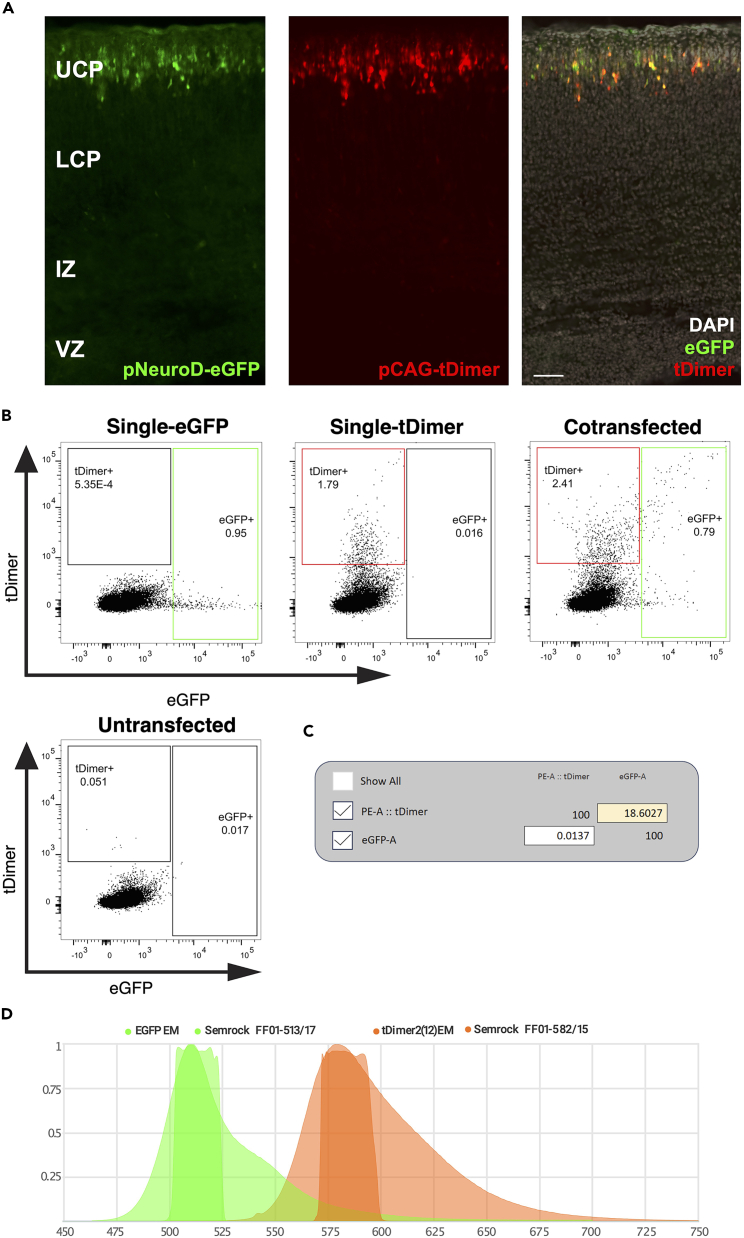
Gating strategy for separating a cell population into subpopulations by flow cytometry based on the transfection of the construct pNeuroD-eGFP/pCAG-tDimer (A) Coronal sections of the murine somatosensory cortex of embryonal age E18 after IUE at E14 showing fluorescent protein expression in the target cell population: pNeuroD-eGFP expressed in post-mitotic neurons (**left**), pCAG-tDimer expressed ubiquitously (**middle**) and merged (**right**). UCP: upper cortical plate, LCP: lower cortical plate, IZ: intermediate zone, VZ: ventricular zone. Scale bar: 100 μm. (**B) Singlets SSC populations** of both single transfected controls, untransfected, and cotransfected samples with the construct pNeuroD-eGFP/pCAG-tDimer. The FMO control of the single eGFP transfection and the FMO control of the single tDimer transfection, together with the untransfected control, were used to calculate the compensation. Compensation with the FMO signals brighter than the signal in the cotransfected sample ensured correct differential fluorescence detection in the cotransfected sample. This compensation and gate setting served as template every time the cotransfected samples were sorted. **Note**: Based on our experimental question, eGFP+ cells may also have tDimer fluorescence (double positive). This experiment was performed on 20,000 cells of E18 embryonic cortices. (C) Computational compensation matrix (“Compensation Wizard” of BD FACS Diva software; recreated with FlowJo) defined values for compensation of spillover into each other BP filter. (D) The BP filters 582/15 and 513/17 covered well the peaks of the emission spectra of the fluorescent proteins used here, tDimer and eGFP, respectively. The plot was generated with FPbase.^17^

**Figure 7:**
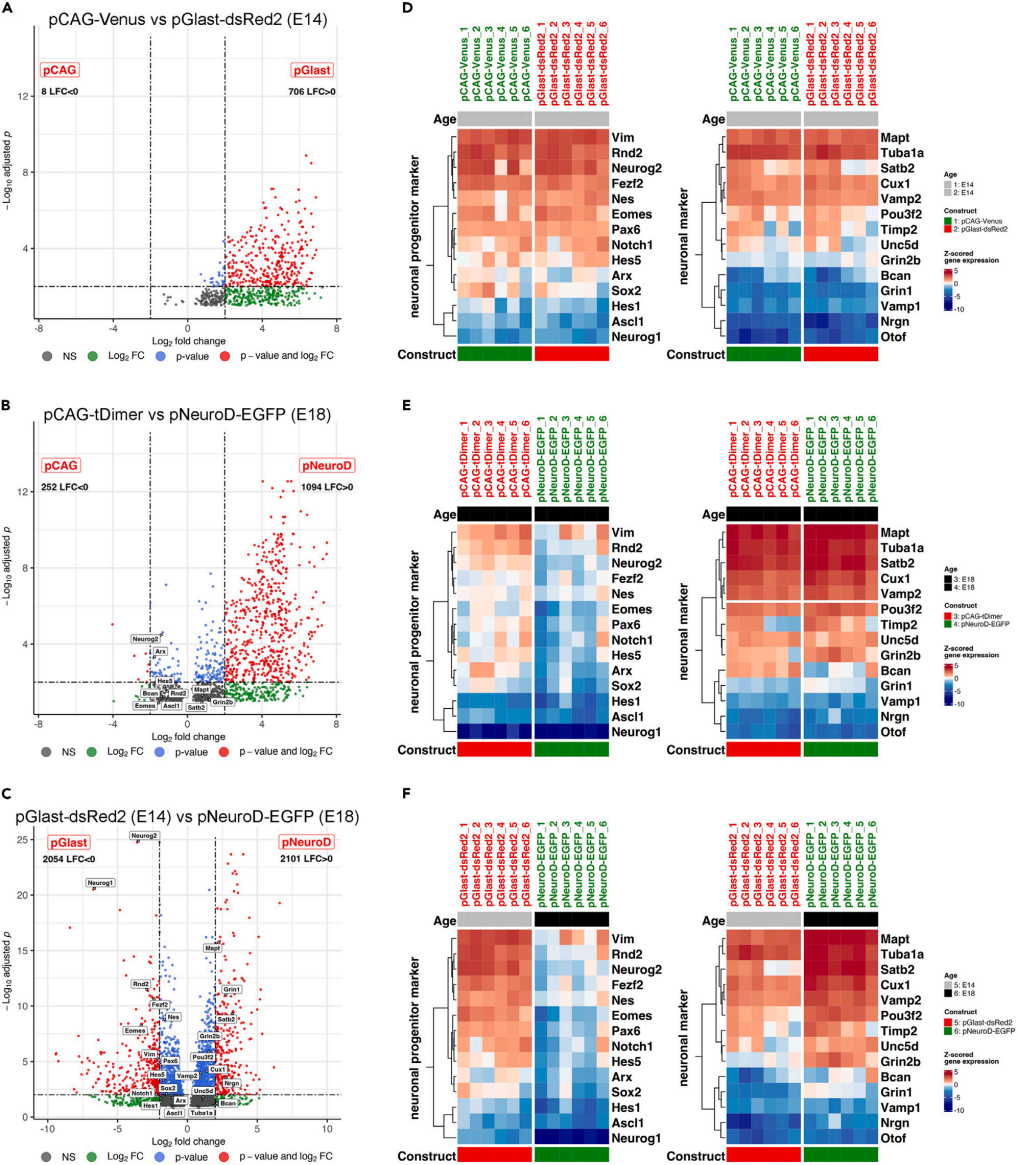
Transcriptional analysis in progenitors and developing neurons reveal differentially expressed genes (DEGs) and enrichment for developmentally regulated marker gene expression in specific progenitor and neuronal subpopulations (A–C) Flow cytometry-based isolation of cell populations sorted by fluorescent proteins driven by developmentally active promoters show enrichment for characteristic genes of the respective targeted cell identity (white boxes) after bulk RNA-seq. A) At E14, DEGs were detected in pGlast-dsRed2 positive progenitors compared to pCAG-Venus positive controls, but no enrichment for characteristic genes of progenitor cell identity was observed. B) DEGs were detected in isolated pNeuroD-eGFP positive immature neurons compared to pCAG-tDimer positive controls at E18. A volcano plot shows enrichment of multiple individual genes involved in neuronal developmental indicating enrichment of characteristic genes of early neuronal cell identity (white boxes). c) DEGs were detected in pGlast-dsRed2 positive progenitors (E14) compared to pNeuroD-eGFP positive developing neurons (E18). A volcano plot shows enrichment of multiple individual genes indicating enrichment of characteristic genes of progenitor cell identity in pGlast-dsRed2 positive cells (E14) and enrichment of several individual genes involved in neuronal development in pNeuroD-eGFP positive immature neurons (E18), confirming progenitor and neuronal cell identity, respectively (white boxes). Gray = expression not significantly altered, green = Log_2_ fold change (FC), blue = p-value, red = Log_2_ fold change and p-value: Genes plotted in red passed the p.adj. cut-off of <0.01 and the Log_2_ FC cut-off >2.00. (D–F) Commonly used marker genes for progenitor (left) and developing neuronal cells (right), respectively, identified in bulk RNA-seq validate differences in cell identity of isolated cells labeled with D) pCAG-Venus vs. pGlast-dsRed2, E) pNeuroD-eGFP vs. pCAG-tDimer or f) pGlast-dsRed2 vs. pNeuroD-eGFP at time points E14 (gray bars) and E18 (black bars). References to the common usage of these genes in RNA-seq analyses are stated in the expected outcomes section. To facilitate comparison, pGlast (E14) and pNeuroD (E18) samples (in F) compare the same input from the cotransfected cell populations, which were independently scored against one another. Z-score was applied on normalized counts (using the DESeq2::rlog method); heatmap colors range from red (high expression), to white (moderate expression) to blue (low expression).

**Figure 8:**
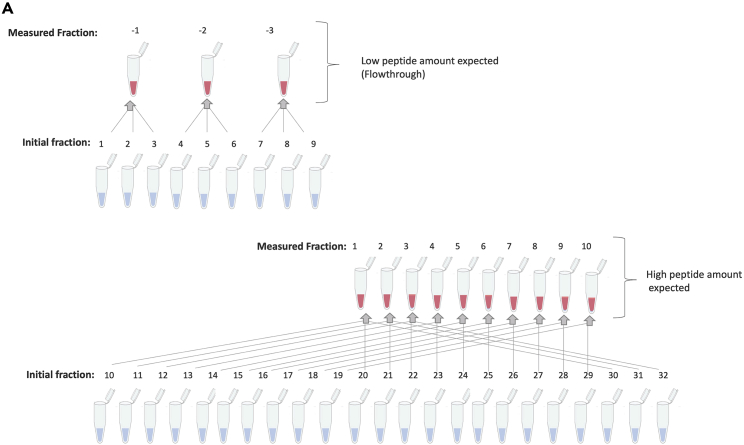
Combination scheme of collected TMT-labeled peptide fractions from basic reversed-phase (RP) chromatography

We routinely perform the different methods described here in detail in our laboratories,^1^^,^^2^^,^^3^^,^^4^^,^^5^^,^^6^ including *in* *utero* electroporation (IUE, Figures 1, 2, and 3), primary cortical cell preparation, flow cytometry (Figures 4, 5, and 6), NGS methods (Figure 7), TMT-labeling, MS sample preparation, quantitative LC-MS/MS (Figure 8), and subsequent bioinformatic analyses. Although we have performed these methods exclusively on mouse cortices in our laboratory, this protocol is probably also applicable to rat cortices, as IUEs in rats are widely established.^7^^,^^8^ Also, refer to other protocols for variations.^9^^,^^10^^,^^11^^,^^12^^,^^13^

**CRITICAL:** Before starting *in utero* electroporation experiments and receiving the necessary training, authorizations and permits are mandatory according to local regulations, which are listed as Institutional permissions.

### Institutional permissions

Perform all mouse experiments according to the local (German and European) Animal Welfare Act and with the approval of local authorities (City-state of Hamburg; Behörde für Justiz und Verbraucherschutz der Freien und Hansestadt Hamburg, Lebensmittelsicherheit und Veterinärwesen) as well as the ethics committee for animal welfare (City of Hamburg).

Breed experimental animals in the central animal facility (ZMNH, UKE, Hamburg, Germany) or provided by a commercial breeder (Charles River, Sulzfeld, Germany). We implement Part B of Directive 2010/63/EU regarding the number of animals housed and the equipment placed in each type II long cage. The housing conditions correspond to the legal requirements in all aspects mentioned in provisions of Appendix A of the EU Directive 2010/63.

In addition to obtaining institutional authorization, the husbandry conditions for pregnant animals are crucial for the subsequent successful yield of an appropriate number of cells:

1. Animal housing for timed mating.
  a. Transfer the animals to the experimental animal facility. Allow them to adapt to the new environment for ≥ 2 weeks. House female and male mice in separate scantainers.
  b. Air-condition the animal rooms (>15 times air exchange, temperature between 20°C–25°C and 45%–65% relative humidity) and set a 12-h light-dark cycle. The animals should have access to food and water *ad libitum.*
  c. Keep male breeders alone in a cage, unless they are kept for mating with one or two females in a separate scantainer intended for mating. However, keep female breeders in groups of no more than five animals, unless they are kept for temporary mating or in groups of no more than three animals after a positive vaginal plug.
    ***Note:*** Perform time matings only in the experimental mouse facility with special permission.
  d. For timed mating, placed the females with the males in the scantainer housing the males in the late afternoon and allow them to mate over the night cycle. In the early morning perform vaginal plug checks.
    ***Optional:*** Females with positive plug tests can be considered E0.5 of pregnancy.
  e. Lift the female at the base of her tail and examine her vaginal opening for a whitish mass to check for a vaginal plug. This mass consists of coagulated secretions from the coagulating and vesicular glands of the male, and as it fills the female’s vagina it persists for 8–24 h after mating.
    ***Optional:*** To improve visibility, spread the labiae slightly with the blunt end of a Graefe Forceps or a Gross Anatomy Probe.
  f. Afterwards, transfer the pregnant females back to the scantainer, housing only females.
2. Pregnant mouse handling.
  a. After a positive vaginal plug test, keep the females with the same plug date in groups and pregnancy is confirmed at E7 at the earliest, followed by daily weight monitoring.^14^ Consider a weight gain of ≥ 1 g at E7 a confirmed pregnancy.
  b. Feed the pregnant females daily with a mixture of apple/banana puree (commercially available baby food) mixed with powdered pellets (Altromin) dissolved in water in a ratio of ∼1/5 puree to dissolved pellets.

***Optional:*** As an additional refinement measure, place mouse biohuts made of tinted transparent polycarbonate in the cage to provide an ideal shelter for mothers to build their nests (Tecniplast). Additionally, provide wooden sticks and cardboard tubes for enrichment.

***Note:*** House the pregnant females transfected with the same constructs in the same cage during the experiment to avoid any external stress factor, such as noise or disturbed circadian rhythm.

***Note:*** Keep the pregnant mice with a maximum of three mother mice per long type II cage.

**CRITICAL:** It is essential to weigh the mice and to adapt mice to soft food at least starting from two days before surgery, to ensure proper weight gain, to calculate pre-operative analgesia on the day of surgery, and to ensure acceptance of post-operative analgesic supply, respectively. If mothers did not gain weight accordingly,^14^ refrain from performing surgery to avoid having a small litter or poorly developed offspring. At E14, a maternal weight gain of at least ∼ 4–5 grams is expected, compared to their weight at E0.5 (vaginal plug day) for 8–10-weeks old C57BL/6J females, which originally weighed ∼ 20–25 grams at E0.5.

### DNA preparation

**Timing: 60 min (for step 3)**

**Timing: 2 days (for step 4)**

3. Preparation of micropipettes for DNA injection.
  a. Pull the borosilicate micropipettes (Science Products GmbH) with the Micropipette puller (P-97 Flaming/Brown MP Puller, Sutter Instruments Co.) with the following parameters.
    ***Note:*** Program #2, Heat - 465, Pull - 50, Velocity - 80, Time - 70.
    ***Note:*** Adjust parameters for other instruments and adjust the settings every time the heating filament of the puller is changed.
  b. Within this time, prepare ∼50–60 micropipettes. Store them in 3 different 15-cm cell culture plates fitted with a stripe of play dough (Figure 1A, Insert).
    ***Note:*** These settings should produce a pipette with a ∼1 cm long tip and a long shoulder that tapers gradually (Figure 2C, Insert). Break off pulled pipettes with forceps at ∼2 mm from the shoulder of the pipettes.
    ***Optional:*** Mark the outer body of the pipettes every 5–10 mm with a waterproof marker, which corresponds to ∼5–10 μL.
4. Preparation of DNA for injection.
  a. Purify plasmids using an EndoFree Plasmid MaxiPrep Kit (QIAGEN).
    ***Note:*** The final DNA concentration, determined using a NanoDrop device (Thermo Scientific), should ideally be higher than 1 μg/μL. Higher DNA concentrations produce higher transgene expression and brighter fluorescence. Higher concentration of shRNA-expressing plasmids ensures sufficient down-regulation of the target protein. The concentration of expression plasmids or shRNA expressing plasmids is optimal at ∼4 μg/μL. For marker proteins such as tDimer, a concentration of as low as 0.1 μg/μL can be sufficient.
  b. Dilute fluorescent marker plasmids to 1–0.1 μg/μL in sterile ddH_2_O.
    ***Note:*** The plasmid mix consists of specific shRNAs expressing plasmids, along with a fluorescence reporter plasmid (e.g., pNeuroD-eGFP, for a neuronal-specific eGFP reporter, pGlast-dsRed2, for an apical radial glial cell-specific dsRed2 reporter).
  c. Add 1/10 - 1/20 volume of 1% Fast Green (Sigma) to the DNA solution as a tracer (final 0.1%–0.05%).
    ***Note:*** To discriminate between control and experimental groups within one litter, use fluorescent reporters (e.g., eGFP or RFP reporters) that are distinguishable for late embryonic and postnatal ages.
    **CRITICAL:** The promoter driving the expression of the fluorescent marker protein is imperative for flow cytometry-based isolation of distinct neuronal cohorts after shRNA-based downregulation of the target protein. Developmentally active promoters are specific for distinct cell types, such as pGlast in apical radial glial cells (aRGCs), and pNeuroD in early postmitotic neurons.^15^^,^^16^ The here so-called “promoter” element has additional synthetic elements such as the CMV early enhancer element for pCAG, or an IRES sequence in case of pNeuroD to reliably express the respective fluorescent protein. For more details, see paragraph 'expected outcomes'.

### Preparations for flow cytometry experiments

**Timing: 4–6 h**

The Fluorescence-Activated Cell Sorting (FACS) is an operator-dependent procedure. A core facility with trained staff might be required. Flow cytometer run with the BD FACSDiva software need to be prepared before starting.

5. Set up flow cytometer.
  a. Select the adequate nozzle size (100 μm) and set up the correct configurations including performance of the Accudrop to calculate proper drop delay (Figures 4A and 4B).
  b. Before starting the sort of the cells of interest (Figures 5A and 6A), select the appropriate laser and filter combinations to detect the fluorescent proteins transfected via IUE. The blue laser will be needed to detect the eGFP and Venus fluorescence. The blue laser excites at 488 nm, the emitted light for eGFP (emission maximum 507 nm) is detected by a band pass filter 513/17 nm, the emitted light for Venus (emission maximum 528 nm) is detected by a bandpass filter 530/30 nm. The green laser will be needed to detect dsRed2 and tDimer fluorescence. The green laser excites at 561 nm. The emitted light for dsRed2 (max. emission 583 nm) is detected by a band pass filter 610/20 nm, whereas the emitted light for tDimer (max. emission 579 nm) is detected by a band pass filter 582/15 nm.
    **CRITICAL:** Check for optimal coverage of the filters to the used fluorochrome, e.g. using FPbase,^17^ as done in Figures 5D and 6D.
  c. Set up **compensation controls.** The compensation controls need to be run for all the different fluorochromes used in the different experimental setups separately. To do this, run the protocol as described below, but this time start with single transfected cells and calculate the compensation matrix e.g., using the “Compensation wizard” of the BD FACS Diva Software. Use untransfected cells as a negative control.
    ***Note:*** Non-transfected cells should also originate from cortical tissue, but from non-electroporated embryos.
  d. Fluorescence Minus One (FMO) controls using cell suspensions from brains transfected with only a single fluorophore (Figures 5B and 6B) allow initial gate setting. Note: Consider the scattering of background signal of the negative population for a given fluorophore emission while using a flow analysis panel with two or multiple fluorochromes. Next, create a template for the flow cytometric workflow to use in the further sorting workflow:
6. Design template for sorting.
  a. Run your cell suspension. Identify the cells by size and granularity in the Forward Scatter Area (FSC-A) versus Side Scatter Area (SSC-A) (Figure 4B).
  b. Exclude debris and laser noise – all of which have a low FSC value – from the analysis. Initial gating with non-transfected cells (Figures 5B and 6B, bottom panels) helps to set and adjust the FSC threshold as strictly as possible. Use this gate for the next scatter plot.
  c. From this identified scatter population create two doublet exclusion plots: An FSC-Area (FSC-A) versus FSC-Width (FSC-W) plot and again by using this population an SSC-A versus SSC-W plot to exclude any remaining doublets (Figure 4B). Doublets or multiples show higher area and width signals compared to single cells, whereas the height remains almost the same. Thus, use a mismatch between height, width, and area to identify and exclude them from the analysis.
  d. Place the prepared cell suspension with the double transfected cells to the flow cytometer, acquire 5,000 events and create from the single cells the sorting gates (the sort layout). Lastly, create gates that contain the cells enriched for the two channels needed to identify the fluorescent proteins of interest (Figures 5B and 6B).

***Note:*** Reuse the template for your sorting strategy. However, slightly adjust the gates that select the target cell populations for each experiment. For more details, see paragraph 'expected outcomes'.

## Key resources table

**Table:** 

REAGENT or RESOURCE	SOURCE	IDENTIFIER
**Chemicals, peptides, and recombinant proteins**		

Vidisic eye gel	Bausch and Lomb	N/A
Iodobromine/ Braunoderm	Braun	Cat#3881105
Fast Green	Sigma	Cat#F7252
TEMGESIC 0.3 mg (buprenorphine)	Indivior Europe Ltd. Dublin, Ireland	Cat#00345928
Metacam (Meloxicam)	Boehringer Ingelheim	Cat#158669-002
Isoflurane Baxter vet	Baxter	Cat#HDG9623V
DPBS, no calcium, no magnesium	Invitrogen	Cat#14190094
Hibernate-E medium	Thermo Fisher Scientific	Cat#A1247601
HBSS	Invitrogen	Cat#14170088
Papain	Sigma	Cat#P4762
DNaseI	Sigma	Cat#D4263
FCS	Biochrom	Cat#S0115
DMEM	Invitrogen	Cat#6196526
Trypan blue	Invitrogen	Cat#15090046
Hoechst dye	Invitrogen	Cat#33258; RRID: AB2651133
Triethyl ammonium bromide (TEAB)	Sigma-Aldrich	Cat#241059
Sodium deoxycholate (SDC)	Sigma-Aldrich	Cat#30970
Dithiothreitol (DTT)	Sigma-Aldrich-Merck	Cat#DTT-RO
Iodoacetamide (IAA)	Sigma-Aldrich	Cat#I1149
Powdered trypsin (sequencing grade modified trypsin)	Promega	Cat#V5111
Pierce HeLa protein digest standard	Thermo Fisher Scientific	Cat#88328
Formic acid (FA)	Promega	Cat#F0507-100ML
Hydroxylamine	Sigma-Aldrich	Cat#438227
Ammonium bicarbonate	Sigma-Aldrich	Cat#A6141
Acetonitrile	Sigma-Aldrich-Merck	Cat#271004-100ML

**Critical commercial assays**		

QIAGEN Endo-Free Maxi Prep Kit	QIAGEN	Cat#12362
TMT10plex Reagent Set for Isobar Marking	Thermo Fisher Scientific	Cat#90110
RNeasy Micro Kit (50)	QIAGEN	Cat#74004
Eukaryote Total RNA Pico assay	Agilent	Cat#5067-1513
SMART-Seq v4 Ultra Low Input RNA Kit	Clontech Laboratories	Cat#634891
Nextera XT DNA Library Preparation Kit	Illumina	Cat#FC-131-1096

**Experimental models: Organisms/strains**		

Mice; C57BL/6J; both sexes, preferentially < one year old	Charles River	https://www.bionity.com/en/companies/24848/charles-river-germany-gmbh-co-kg.html; Strain #: 632

**Recombinant DNA**		

pSilencer2-U6-shControl	Xie et al.^18^	N/A
pSilencer2-U6-shCep120	Xie et al.^18^	N/A
pCAG-tDimer	A gift from Thomas Oertner	N/A
pCAG-Venus	A gift from Phil Sharp; (de Anda et al., 2010^19^)	RRID: Addgene_127346
pGLAST-dsRed2	A gift from Nicholas Gaiano (via Addgene); (Mizutani et al.)^16^	RRID: Addgene_17706
pNeuroD-ires-eGFP	A gift from Zhigang Xie; (de Anda et al., 2010^19^) and via Addgene	RRID:Addgene_61403

**Software and algorithms**		

Human Reviewed FASTA Database	UniProt	N/A
SEQUEST algorithm integrated into Proteome Discoverer 2.4	Thermo Fisher Scientific	https://www.thermofisher.com/de/de/home/industrial/mass-spectrometry/liquid-chromatography-mass-spectrometry-lc-ms/lc-ms-software/multi-omics-data-analysis/proteome-discoverer-software.html
R software environment	R Core Team, 2022	https://cran.r-project.org/
rstatix (CRAN R package)	Kassambara, 2021^20^	https://cran.r-project.org/web/packages/rstatix/index.html
ggplot2 (CRAN R package)	Warnes et al., 2022^21^	https://cran.r-project.org/web/packages/gplots/index.html
dendextend (CRAN R package)	Galili, 2015^22^	https://cran.r-project.org/web/packages/dendextend/index.html
DESeq2 v. 1.43.0 (Bioconductor R package)	Love et al., 2014^23^	https://bioconductor.org/packages/devel/bioc/vignettes/DESeq2/inst/doc/DESeq2.html
clusterProfiler (Bioconductor R package)	Wu et al., 2021^24^	https://bioconductor.org/packages/release/bioc/html/clusterProfiler.html
STRING	Szklarczyk et al., 2019^25^	https://string-db.org/
WebGestalt	Liao et al., 2019^26^	http://www.webgestalt.org
ComplexHeatmap v. 2.14.0	Gu et al., 2016^27^	https://doi.org/10.1093/bioinformatics/btw313
dplyr v. 1.0.10	Wickham, 2019^28^	https://dplyr.tidyverse.org
mixOmics v. 6.17.12	Rohart et al., 2017^29^	https://CRAN.R-project.org/package=mixOmics
tidyverse 1.3.2	Wickham, 2019^28^	https://doi.org/10.21105/joss.01686
BD FACSDiva software	BD Biosciences	https://www.bdbiosciences.com/en-us/products/software/instrument-software/bd-facsdiva-software
FlowJo	BD Biosciences	https://www.bdbiosciences.com/en-us/products/software/flowjo-v10-software

**Other**		

Mouse food powder	Altromin	Cat#1311P
Mouse biohuts made of tinted transparent polycarbonate	Tecniplast	N/A
Graefe forceps	Fine Science Tools	11049-10
Gross anatomy probe	Fine Science Tools	10088-15
Micropipette puller	Flaming/Brown MP puller, Sutter Instrument Co.	Model P-97
NanoDrop	Thermo Fisher Scientific	https://www.thermofisher.com/de/de/home/industrial/spectroscopy-elemental-isotope-analysis/molecular-spectroscopy/uv-vis-spectrophotometry/instruments/nanodrop/instruments.html#nanodrop-eight
Borosilicated micropipettes	Science Products GmbH	Cat#GB100TF
Isoflurane-O2 mixer device, Dräger Vaporizer 19.1	Drägerwerk AG	N/A
Fluovac	Harvard Apparatus	N/A
Isoflurane adsorber	Stoelting	Cat#50207
Double edge PERSONNA coated razor blades	Electron Microscopy Sciences	Cat#72000
Ring forceps – 9 cm	Fine Science Tools	Cat#11106-09
Standard pattern forceps – serrated straight 14 cm	Fine Science Tools	Cat#11000-14
Strabismus scissors – straight blunt 9 cm	Fine Science Tools	Cat#14074-09
Kelly hemostat – serrated straight 14 cm	Fine Science Tools	Cat#13019-14
Forceps – serrated straight 7cm	Fine Science Tools	Cat#11064-07
Dumont #5 mirror finish forceps	Fine Science Tools	Cat#11252-23
Extra thin Iris scissors – straight 10.5 cm	Fine Science Tools	Cat#14088-10
Extra fine Iris scissors – curved 8.5 cm	Fine Science Tools	Cat#14085-08
Strabismus scissors straight blunt 9 cm	Fine Science Tools	Cat#14074-0908
Surgical scissors Mayo-Stille – 17 cm	Fine Science Tools	Cat#14012-17
Alcohol pads	Braun	Cat#9160612
Platinum tweezer electrode 5 mm	Harvard Apparatus	Cat#EC1 45-0489
Electroporator ECM 830 BTX and BTX generator footswitch (model 1250FS)	Harvard Apparatus	Cat#EC1 45-0052
Vicryl thread	Ethicon	Cat#V994H
Silk thread	Silkam	Cat#C0762130
Stereo microscope model SZX16	Olympus	https://www.olympus-ims.com/en/microscope/szx16/
BD FACS AriaIIIu	BD Biosciences	N/A
BD FACS AriaFusion	BD Biosciences	N/A
ThermoMix C	Eppendorf, Germany	Cat#5382000015
Probe sonicator	N/A	N/A
SpeedVac SC110 Savant	Thermo Fisher Scientific	N/A
Monolith column ProSwift RP-4H, 1 mm × 250 mm	Thermo Fisher Scientific	Cat#164921
Agilent 1200 series HPLC monolith column ProSwift RP-4H, 1 mm × 250 mm	Agilent Technologies Thermo Fisher Scientific	https://www.agilent.com/en/products/liquid-chromatography
RP fractionation column Agilent 1200 series HPLC	Agilent Technologies	https://www.agilent.com/en/products/liquid-chromatography
Dionex Ultimate 3000 UPLC	Thermo Fisher Scientific	https://www.thermofisher.com/de/de/home/industrial/chromatography/liquid-chromatography-lc/hplc-uhplc-systems
Acclaim PepMap 100, 75 μm × 50 cm, 100 Å pore size , 2 μm particle size	Thermo Fisher Scientific	Cat#164564
Orbitrap Fusion	Thermo Fisher Scientific	https://www.thermofisher.com/de/de/home/industrial/mass-spectrometry/liquid-chromatography-mass-spectrometry-lc-ms/lc-ms-systems
7900HT fast real-time PCR system	Thermo Fisher Scientific	Cat#4329001
2100 Bioanalyzer instrument	Agilent	Model G2939B
Illumina NextSeq 550 platform	Illumina	https://emea.illumina.com/systems/sequencing-platforms/nextseq.html

## Materials and equipment

• Micropipette puller: Flaming/Brown MP Puller Model P-97 (Sutter Instruments Co.)
• Electroporator: ECM 830 BTX (Harvard Apparatus).
• Flow cytometry: AriaIIIu and/or AriaFusion (BD).

***Alternatives:*** MoFlo (Beckman Coulter) and Cell Sorter SH800S (Sony).

• HPLC system: Agilent 1200 series (Agilent Technologies).

***Alternatives:*** Vanquish Core HPLC Systems (Thermo Scientific, Bremen, Germany), UltiMate 3000 UHPLC system (Thermo Fisher Scientific).

• HPLC monolith column: ProSwift RP-4H, 1 mm 3 250 mm (Thermo Fisher Scientific, Bremen, Germany).

***Alternatives:*** 100 mm × 1.0 mm Acquity (BEH C18 column (Waters, Milford, USA), Zorbax 300 Extend-C18 column (3.5 μm, 4.6 × 250 mm; Agilent, Santa Clara, USA).

• nano-UPLC system: Dionex Ultimate 3000 (Thermo Fisher Scientific, Bremen, Germany).

***Alternatives:*** nano-flow UPLC EASY nLC 1200 (Thermo Scientific), Evosep One (Evosep, Odense, Denmark).

• nano-UPLC trapping column: Acclaim PepMap 100, 100 μm × 2 cm, 100 Å pore size, 5 μm particle size (Thermo Fisher Scientific, Bremen, Germany).

***Alternatives:*** nanoEASE M/Z Symmetry C18, 180 μm × 2 cm, 100 Å pore size, 5 μm particle size (Waters, Milford, USA). For loading, the flow rate must be changed to 15 mL/min from 5 mL/min (Compared to Acclaim PepMap 100).

• nano-UPLC analytical column: Acclaim PepMap 100, 75 μm × 50 cm, 100 Å pore size, 2 μm particle size (Thermo Fisher Scientific, Bremen, Germany)

***Alternatives:*** ACQUITY UPLC Peptide CS C18, 75 μm × 25 cm, 130 Å pore size, 1.7 μm particle size (Waters, Milford, USA) (For loading, the flow rate must be changed to 15 mL/min from 5 mL/min (Compared to Acclaim PepMap 100).

• Mass spectrometer: Orbitrap Fusion Quadrupole-Iontrap-Orbitrap Tribrid mass spectrometer (Thermo Fisher Scientific).

***Alternatives:*** Orbitrap Lumos Quadrupole-Iontrap-Orbitrap Tribrid mass spectrometer (Thermo Fisher Scientific), Orbitrap Eclipse Quadrupole-Iontrap-Orbitrap Tribrid mass spectrometer (Thermo Fisher Scientific), Orbitrap Ascend Quadrupole-Iontrap-Orbitrap Tribrid mass spectrometer (Thermo Fisher Scientific).

**Table:** Trypsin stock solution (see **Step 3.a**)

Reagent	Final concentration
Triethyl ammonium bicarbonate (TEAB)	100 mM
Sodium deoxycholate (SDC)	1%
Trypsin	0.5 mg/mL

Dissolve in ddH_2_O, pH 8.5, store at −20°C and do not store for more than 2 weeks.

***Note:*** Trypsin is highly active between pH 8.0 and 10.0. Any MS-compatible buffer that fulfills this criterion can be used for the reconstitution of powdered trypsin. The lysis buffer (0.1 M TEAB with 1% SDC, has a pH of 8.5 and is hence well suited for tryptic digestion.

**CRITICAL:** (Step-by-step method details for ‘RNA-seq or Quantitative LC-MS/MS’: Proteomic sample preparation) Sodium deoxycholate (SDC) is harmful and classified under hazard statement H302.

**CRITICAL:** (Step-by-step method details for ‘RNA-seq or Quantitative LC-MS/MS’: Proteomic sample preparation) Dithiothreitol (DTT) is corrosive and classified under hazard statement H318.

**CRITICAL:** (Step-by-step method details for ‘RNA-seq or Quantitative LC-MS/MS’: Proteomic sample preparation) Iodoacetamide (IAA) is toxic and classified under hazard statements H301, H315, H317, H319, H334, and H413.

**CRITICAL:** (Step-by-step method details for ‘RNA-seq or Quantitative LC-MS/MS’: Proteomic sample preparation and Step-by-step method details for ‘RNA-seq or Quantitative LC-MS/MS’: LC-MS3 measurement) Formic acid (FA) is toxic, corrosive, and flammable and classified under hazard statements H226, H302, H314, H318, H331, and H402.

**CRITICAL:** (Step-by-step method details for ‘RNA-seq or Quantitative LC-MS/MS’: Tandem Mass Tag (TMT) 10 plex labelling, Step-by-step method details for ‘RNA-seq or Quantitative LC-MS/MS’: Offline basic Reversed Phase (RP) HPLC fractionation, and Step-by-step method details for ‘RNA-seq or Quantitative LC-MS/MS’: LC-MS3 measurement) Acetonitrile is flammable, harmful, and classified under hazard statements H225, H302 + H312 + H332, and H319.

**CRITICAL:** (Step-by-step method details for ‘RNA-seq or Quantitative LC-MS/MS’: Tandem Mass Tag (TMT) 10 plex labelling) Hydroxylamine is a health hazard, corrosive, and harmful to the environment and is classified under hazard statements H290, H302 + 312, H315, H317, H319, 351, 373, and H400.

**CRITICAL:** (Step-by-step method details for ‘RNA-seq or Quantitative LC-MS/MS’: Offline basic Reversed Phase (RP) HPLC fractionation) Ammonium bicarbonate is harmful and classified under hazard statement H302.

## Step-by-step method details

### *In utero* electroporation

**Timing: 60 min per mouse (in total)**

**Timing: 40 min (for step 1)**

**Timing: 10 min (for step 2)**

**Timing: 10 min (for step 3)**

This procedure allows the transfection of plasmid constructs to express fluorescent reporters in specific cellular cohorts and the downregulation of protein expression via specific shRNAs in the same cells during mouse brain development.

1. Analgesics and induction of anesthesia and other preparations.
  a. Autoclave the dissection instruments (1× Strabismus scissors straight blunt 9 cm, 2× ring forceps, 2× Standard Pattern Forceps – Serrated Straight 14 cm, Extra Thin Iris Scissors – Straight 10.5 cm, and Extra Fine Iris Scissors – Straight 8.5 cm, Kelly Hemostat – Serrated Straight 14 cm – purchased from Fine Science Tools) the day before the experiment.
  b. Cut all lab tapes and gauze and arrange surgical tools beforehand (Figure 1A).
  c. Sterilize the working area, cold source lamps and platinum paddles of the tweezers-type electrodes with 70% ethanol before setting up the mouse for anesthesia.
  d. Set up the electroporator. Use 35 V, 50 ms On, 950 ms Off, 5 pulses for E14 or E15 embryos.
    ***Note:*** For younger or older animals, the voltage should decrease or increase accordingly. Use a 1–2 V increment for each day. The current flow is activated by a foot switch.
  e. Anesthetize the mice in an isoflurane chamber at least 30 min before the surgical procedure. Administer buprenorphine according to their weight into the neck skin (0.1 mg/kg body weight s.c.) to relieve pain. Return the mice to their home cage for 30 min until the analgesia is fully effective.
    ***Note:*** Carry out all analgesia procedures according to internationally established protocols and in accordance with the recommendations of the German and American neuroscientific societies. Advice and approval from local animal welfare, ethical and veterinary committees are mandatory. Follow the analgesia protocols according to the local legislation regarding the use of opioid drugs and animal welfare.
2. Laparotomy.
  a. Before starting the surgery, anesthetize the mouse again in an isoflurane chamber. Cover the eyes of the mouse with an optical lubricant gel, e.g., Vidisic gel (Bausch and Lomb) to prevent the cornea from drying out during the surgery process (Figure 1B).
  b. Quickly remove the mouse from the anesthesia chamber and fix it with lab tape on its back, dorsal decubitus, to a heating pad (maintained at ∼37°C) on a well-lit operating table. Place the nose of the mouse in a ventilator tube/nozzle for a constant supply of isoflurane in oxygen (0.65 L/min O_2_ flow) for anesthesia throughout the surgery (Figure 1C). Set the isoflurane flow rate to 4% for anesthesia induction and to ∼1.5%–2% for maintenance during the *in utero* electroporation.
  c. Assess complete analgesia (surgical tolerance) by checking the absence of a reflex response to pinching the skin between the toes with forceps (interdigital reflex). This method is considered the most sensitive indicator of complete analgesia.^30^
    ***Note:*** The isoflurane-O_2_ mixed air coming out of the isoflurane chamber and any excess isoflurane-O_2_ mixed air passing through the ventilator tube/nozzle is passed through the activated charcoal isoflurane adsorber (Stoelting) to prevent releasing any isoflurane into the surroundings.
    **CRITICAL:** Since isoflurane suppresses breathing, monitor the breathing of the mice closely during anesthesia. Each mouse responds differently to anesthesia; therefore, frequently check the flow of isoflurane administered and adjust the concentration accordingly during surgery to ensure adequate respiration.
  d. Disinfect the abdomen with sterile 70% ethanol and shave the fur over the surgery region using a double-edged coated razor blade (Electron Microscopy Science) using shaving foam (commercially available).
  e. Sterilize the skin with sterile 70% ethanol and wipe with Iodobromine (Braunoderm B. Braun) using a cotton swab in a downward motion. Avoid rubbing back and forth to prevent cross-contamination from the genital area and to maintain sterile conditions.
  f. Use Fine Iris scissors to cut the skin longitudinally for about 2 cm along the midline/ Linea alba. Do not cut the midline directly (which appears as a thin white line) to improve the chances of good healing (Figure 2A).
  g. Separate skin and muscle tissue of the abdomen carefully without tearing up the muscle tissue using Strabismus scissors straight blunt 9 cm.
  h. Make an incision of about 1.5 cm in the muscle of the abdominal cavity.
  i. Cover the abdomen with folded gauze with a ∼3 cm long longitudinal slit in its center.
  j. Moisten the gauze with pre-warmed (∼37°C) sterile saline. Place the falcon tube with the saline in a heated water bath to maintain the temperature.
3. Electroporation.
  a. Hold one side of the incision site with Standard Pattern Forceps – Serrated Straight 14 cm and pull up the skin. Apply excessive pre-warmed saline (∼37°C) to the abdominal cavity – this will help to extract the uterus.
  b. Remove the uterus using ring forceps by gently grasping the uterine horn. It is essential not to damage the placenta or the blood vessels connected with the uterus (Figure 2B). Throughout surgery, keep the abdominal cavity and the uterus moist by repeatedly applying pre-warmed sterile saline.
  c. Carefully hold one uterine horn with serrated forceps and carefully push one embryo with the ring forceps to the uterine wall. In good lightning conditions, the uterine wall and skull are transparent, and the telencephalon and ventricles become visible.
  d. Set up the mouth pipette (Aspirator tube assemblies for calibrated microcapillary pipettes, Sigma): Inset one pulled micropipette into the mouth pipette. Draw about ∼10 μL plasmid solution into the micropipette for all embryos of the same experimental group.
  e. Hold the embryo with the ring forceps or fingers and use the other hand to hold the mouth pipette. Penetrating the neocortex for ∼2 mm in depth with the micropipette, ∼1 mm lateral to the central fissure and ∼0.5–1 mm above an imaginary line between the eyes, until the tip of the micropipette hits one of the lateral ventricles.
    ***Note:*** If you insert the pipette perpendicular to the cortex surface and hold the pipette still during the injection, the brain tissue will hardly be damaged (Figure 2C).
  f. Inject 1–2 μL of the shRNA or DNA plasmid/Fast Green solution into one of the lateral ventricles.
    ***Note:*** If done correctly, the lateral ventricles become visible with plasmid/Fast Green mix and appear in a crescent shape (Figure 2D, white arrowheads).
  g. Continue injecting all embryos in the litter except those near the vagina.
  h. Apply pre-warmed saline to the embryos before and during electroporation – this ensures firm contact between the platinum paddles of the electrodes and the embryo heads to enable sufficient current flow.
  i. Hold the injected embryos along their anteroposterior axis with the ring forceps or with the fingertips and put a 5 mm tweezers-type electrode (Harvard Apparatus) across the brain with the positive electrode above the targeted brain site and the negative pole to the opposite side (Figure 2E).
    **CRITICAL:** Avoid electroporating the embryos closest to the vaginal band to prevent abortions. This is one of the most critical procedures in the entire protocol (see problem 2).
  j. Deliver the electric pulses to the embryo: Five current pulses (50 ms pulse/950 ms interval) at 30 V for E13 and 35 V for E15 are delivered from the Electroporator ECM 830 BTX (Harvard Apparatus), across the heads of embryos using the dipolar platinum tweezers-type electrode.
    ***Note:*** For embryos collected before the delivery, take note on the exact number of embryos on each of the uterine horns and mark the electroporated hemisphere side and embryo position with the specific conditions to trace them back later.
    **CRITICAL:** Avoid moving the embryos and the uterus extensively during the electroporation (Figure 2). Extensive movement will harm the integrity of the embryo and its connection to the placenta, the uterus or the blood vessels connecting the uterus; either of this can increase the risk of an abortion.
  k. Again, apply pre-warmed saline and carefully push the embryos back into the abdominal cavity.
  l. Fill the cavity with warm saline, hold both sides of the incision site with ring forceps, and pull up the skin repeatedly (Figure 2F).
    **CRITICAL:** Fill the abdomen with pre-warmed saline and pull up the skin gently to help the embryos slide back into their original position.
  m. Close the surgical incision in the abdominal wall using a Vicryl absorbent thread (Ethicon), and then the outer skin layer with a Silk thread (Silkam) by continuous closure with looped sutures.
  n. Apply iodobromine (Braun) thoroughly to the sutured site to prevent post-operative infections.
  o. Keep the animal warm at 37°C on a heating pad and under surveillance until complete recovery from anesthesia and retain the mouse cage on a heating pad for 48 h post-surgery.
4. Postoperative analgesia therapy.
  a. After the surgery, inject meloxicam subcutaneously once (5 mg/kg body weight s.c.).
    ***Alternatives:*** Other non-steroidal anti-inflammatory drugs (NSAIDs) affecting COX-1 and COX-2 inhibitors, e.g., carprofen, flunixin-meglumine, ibuprofen, etodolac or diclofenac are acceptable (GV-SOLAS, Committee on Anesthesia, Pain Management in Experimental Animals, September 2020). Please check the regulations issued by your local authorities.
  b. For three days post-surgery, give meloxicam to the animals orally (10–20 mg/kg body weight, twice daily) mixed in soft food to which the animals were accustomed a few days before the surgery.
  c. In case of reduced food intake, switch back the analgesia protocol to parenteral analgesia (meloxicam, 20 mg/kg body weight s.c). In case of severe pain, administer buprenorphine (0.1 mg/kg body weight s.c., 3× daily). These measures are according to the recently updated recommendations of GV Solas for administering analgesics in experimental animals. Stick to the respective score sheet for the experiment and consider humane endpoints (see problem 1).
    **Pause point:** until cell collection.
    ***Note:*** After electroporation, flow cytometry experiments can be performed after 1.5 days *in utero*. In general, the expressed fluorophore is stable in the mouse brain for at least 4 weeks. Harvest the cells within this time frame depending on your experimental question.

### Flow-cytometry-based isolation of cells from developing mouse cortices

**Timing: <4 h(in total)**

**Timing: <2 h per mouse (for step 5)**

**Timing: 2 h (for step 6)**

5. Cortical neural cells preparation.
  In this step, the transfected cortical regions are firstly dissected and subsequently dissociated to obtain a single-cell suspension for subsequent sorting by flow cytometry.
  **CRITICAL:** To minimize the time (which should not be more than 2 h) between sacrificing the animal and taking the cells to flow cytometry, set up all required materials before starting (Figure 3A).
  a. Autoclave the dissection instruments (1× Surgical scissors Mayo-Stille – 17 cm, Standard Pattern Forceps – Serrated Straight 14 cm, 1× Extra Thin Iris Scissors – Straight 10.5 cm, 1× Extra Fine Iris Scissors – Curved 8.5 cm, 1× Forceps - Serrated Straight 7 cm, and 2× Dumont #5 Mirror Finish Forceps –- purchased from Fine Science Tools) and sterilize the work area with 70% ethanol beforehand.
  b. Euthanize the pregnant mouse with the CO_2_/O_2_ in the cage, decapitate the mouse with surgical scissors Mayo-Stille – 17 cm, and sterilize the abdomen with ethanol.
  c. Cut through the skin and open the abdominal wall using another 1× Extra Thin Iris Scissors – Straight 10.5 cm. Remove the two horns of the uterus and put them into a petri dish using a standard Pattern Forceps – Serrated Straight 14 cm.
  d. Open the uterus carefully and remove the embryos from their amniotic sac and placenta.
  e. Decapitate the embryos with Extra Thin Iris Scissors – Straight 10.5 cm and transfer the heads to a fresh petri dish filled with Hibernate-E Medium (Thermo Fisher) on ice.
    **CRITICAL:** If two or more experimental groups per animal were transfected, consider the order of injection and segregate groups while removing the uterine horns. Collect embryos of different experimental groups in separate petri dishes to avoid mix-ups.
  f. Dissect the whole brains using a pair of Extra Fine Iris Scissors – Curved 8.5 cm and Forceps – Serrated Straight 7 cm in a fresh petri dish containing Hibernate-E Medium (Thermo Fisher) on ice (Figure 3B).
  g. Dissect the cerebral cortex after removal of the skull, meninges and hippocampus according to the classic Banker method.^31^^,^^32^ Strip away the meninges and hippocampi using a pair of Dumont #5 Mirror Finish Forceps, and collect the dissected cortical hemispheres in a well of a 24-well plate (Figure 3C).
  h. Identify brains with noticeable fluorescence visible in the transfected hemispheres under a stereo microscope (Olympus SZX16) equipped with a UV light source (Figures 3D and 3E).
  i. Dissect transfected (fluorescence-positive) cortical regions under the stereo microscope equipped with a UV light source in a Hibernate-E medium on ice.
  j. Pool the isolated cortical pieces from the same experimental group obtained from several brains from the same litter and transfer them into a fresh 15 mL falcon tube containing 1× HBSS (Invitrogen) on ice.
  k. Incubate the tissue pieces in 2 mL 1× HBSS (Hank’s Balanced Salt Solution) with 25 μL of 25 mg/mL stock solution papain (Sigma) and DNaseI 20 μL of 1 mg/mL stock solution (Sigma) for 5–10 min at 37°C.
  l. Add 2 mL 10% FCS (Biochrom) in DMEM (Invitrogen) to stop the digestion reaction, then wash with 1× HBSS, alternatively replacing it with FACS buffer (containing 0.2 mM EDTA in calcium and magnesium free PBS). In general, use HBSS preferentially pre sorting and FACS buffer post sorting to collect the cells in 1.5 mL Eppendorf tubes.
  m. Triturate the cells using a fire-polished Pasteur pipette with a 1 mm opening in 2 mL 1× HBSS 10–20 times thoroughly but gently until suspended.
    **CRITICAL:** Stop triturating once dissociated – mechanical stress on the cells affects the flow cytometry outcome.
  n. Centrifuge the cells at 150 × *g* for 10 min in 2 mL cold 1× HBSS buffer before resuspending the cell pellets in 1 mL 1× HBSS and passing the cell suspensions through a 40 μm insert filter to remove clusters.
  o. Count an aliquot of this cell suspension in a 1:1 or 1:5 dilution with Trypan blue (Invitrogen) using a Neubauer chamber.
    ***Note:*** The recommended range for sorting by flow cytometry per sample is 5 × 10^6^ to 1 × 10^7^ cells per ml. One could downscale the volume based on the cell numbers obtained, e.g. 2.5 × 10^6^ per 0.5 mL or 1.5 × 10^6^ per 0.3 mL. A reduced number of cells affect yields considerably, so it is essential to maintain the recommended range.
    **CRITICAL:** All steps should be performed under sterile conditions in a laminar flow hood. Samples should be kept on 4°C ice. It is helpful to gently agitate the cell suspension every few minutes, as early neurons easily attach to surfaces, which ultimately leads to clogging (problem 4).
6. Cell sorting by flow cytometry.
  Our FACS Facility is located at the Cytometry & Cell Sorting Core Unit, Stem Cell Transplant Clinic, Oncology Center, UKE Hamburg. We used two advanced instruments for sorting by flow cytometry (BD FACS AriaIIIu and BD FACS AriaFusion). Instruments with superior multicolor optics and performance are recommended, with exchangeable nozzles and an in-build automated quality control appropriate for sorting by flow cytometry of the distinct neuronal subpopulations.
  a. Apply the filtered cell suspension to the cytometer. Use the template created earlier.
  b. The nozzle separates the jet into individual droplets.
    ***Note:*** The selection of the nozzle size can be crucial (see problems 3 and 4).
    Only droplets with cells of interest are charged while those out of the sorting gates remain unaffected, even if showing fluorescence.
  c. Laser intersection facilitates the analysis of the cells. Deflection plates attract or repel cells accordingly: Charged droplets are deflected in the electric field and collected into the appropriate container. A single eGFP-positive cell in a single droplet can be an example of a positive charge, while a single RFP-positive cell-containing droplet will have a negative charge.
    ***Note:*** To specifically isolate migrating neurons, include the pNeuroD-eGFP as a reporter plasmid along with shRNA or control plasmids during electroporation. Use appropriate reporter plasmids to isolate different cell types (see the ‘expected outcomes’, Figures 7A–7F and problem 5).
  d. Collect the sorted cells in prepared 1.5 mL eppis already containing 0.5 mL FACS buffer, centrifuge the cells after sorting (150 × *g*, 10 min, 4°C), and remove the excess FACS buffer under sterile conditions. When removing the supernatant, take care not to disturb the cell pellet, which is not visible, and leave about 20 μL in the Eppendorf tubes.
  e. Store the cell pellets at −80°C until further use for up to 24 months.
    **Pause point:** until subjection to downstream experiments.

### RNA-seq or quantitative LC-MS/MS

**Timing: 190 min + 4–16 h (for step 7)**

**Timing: 90 min (for step 8)**

**Timing: 120 min + 90 min QC (for step 9)**

**Timing: 20 h + 4 h QC (for step 10)**

**Timing: 2 h + 4 h sequencing (for step 11)**

Now the sorted cells are subjected to the respective downstream experiments. These range from bulk RNA-seq for transcriptome analyses to LC-MS/MS for proteome analyses and also other experiments that can be performed with frozen, unfixed cells. For more details, see paragraph 'expected outcomes'.

### Sample preparation for mass spectrometry

***Note:*** Proteome analysis, in a bulk/non-single cell setup with the method described below, requires an injection amount of 1 microgram per sample, roughly corresponding to 10,000 fluorescence-positive cells for the selected cell type. Based on the yield from the sort, pool the isolated cells obtained from cortices of 2 or more embryos from the same experimental group to achieve the desired numbers. Novel mass spectrometers can achieve an even higher resolution, so that fewer cells are needed for a comparable outcome.

7. RNA extraction and subsequent sequencing.
  Transcriptome analysis of cell populations in bulk requires 10,000 cells.
  a. Prepare the collected cells with the RNeasy Micro Kit (QIAGEN, #74004) (https://www.qiagen.com/us/Resources/ResourceDetail?id=e112adfa-cc06-4e29-87f8-4820062ae44e&lang=en, last accessed: 31.10.23) according to the manufacturer’s instructions.
  b. Assess RNA quality prior to sequencing with e.g., Agilent 2100 Bioanalyzer using a Eukaryote Total RNA Pico assay.
  c. Due to low amount of material, synthesize cDNA for library generation using the SMART-Seq v4 Ultra Low Input RNA Kit) as per manufacturer’s recommendations (https://www.takarabio.com/a/114896, last accessed: 31.10.23).
  d. Generate the library for sequencing using the Nextera XT DNA Library Preparation Kit.
  e. Then subject the libraries to RNA sequencing on e.g., Illumina NextSeq 550 sequencing platform.
    **Pause point:** until downstream data analyses (Figure 7).
8. Proteomic sample preparation.
  This step ensures the complete extraction and denaturation of proteins from cells. It linearizes heat-denatured proteins through the reduction of disulfide bonds and prevents the reoxidation of thiol groups by alkylation. Moreover, during this step, proteins hydrolyze to peptides after essential amino acids (lysine and arginine). Thereby, they generate at least doubly charged peptides (positive charge at the guanidino group for arginine; positive charge at the amine group for lysine, and N-terminal positive charge) that are relevant for positive mode LC-MS/MS analysis.
  a. Add 100 μL lysis buffer (100 mM triethyl ammonium bicarbonate (TEAB) with 1% (w/v) sodium deoxy**c**holate (SDC, pH 8.5)) to sorted cell collections (1 tube per replicate or sample).
  b. Next, vortex the cell-lysis buffer mixture for 5–10 s on the highest setting.
  c. Lyse the cells for 5 min at 95 °C at a rotation speed of 400 rpm in a ThermoMixC (Eppendorf, Hamburg, Germany).
  d. Again, vortex the cell lysis buffer mixture for 5–10 s on the highest setting.
  e. Use a probe sonicator for six pulses (1 s) at 30% power (PowerPac HC High-Current power supply, Bio-Rad Laboratories, Hercules, USA). This step downsizes DNA, which would otherwise interfere with the MS analysis.
  f. The lysis buffer forms foam during sonication. Therefore, give a quick spin on a Benchtop centrifuge to clear the lysate faster and check the solubility of the pellet. In addition, this step will ensure that all solvent is at the bottom of the tube.
    **CRITICAL:** Do not ultracentrifuge at a high rotation speed for this step, as proteins might sediment.
    ***Note:*** Cell numbers obtained after sorting by flow cytometry are limited. As a rough estimation, 100,000 cells equal 10 μg of protein. Use the entire sample volume for tryptic digestion to minimize the loss of the limited material. Using a proportion of the sample volume for protein concentration determination is not recommended.
    **CRITICAL:** If the cell pellet does not dissolve completely, repeat lysing from last steps of proteomic sample preparation c-f.
  g. Prepare a stock solution of 1 M DTT in lysis buffer. Add DTT to a final concentration of 10 mM. Reduce disulfide bonds at 60°C at a rotation speed of 400 rpm in a ThermoMixC (Eppendorf, Hamburg, Germany) for 30 min.
  h. Prepare a stock solution of 0.5 M IAA in lysis buffer. Add IAA to a final concentration of 20 mM. Reduced disulfide bonds are further reduced at 37°C in the dark at a rotation speed of 400 rpm in a ThermoMixC for 30 min.
    **CRITICAL:** Prepare stock solutions immediately before use and perform the alkylation in the dark. Iodoacetamide is unstable and light-sensitive.
  i. Add 250 ng trypsin, which is 0.5 μL from the trypsin stock solution (see materials and equipment) to the sample.
    ***Note:*** Efficient tryptic digestion can ensure increased accessibility of the amino acid sequence.^33^^,^^34^ A high and comparable digestion efficiency across all analyzed samples additionally ensures accurate quantification from LC-MS/MS data. The digestion efficiency can be analyzed by different methods, including the detailed investigation of the protein-sequence coverage and the cleavage specificity as well as the exponentially modified protein abundance index (emAPI).^35^
    ***Note:*** Trypsin cuts are less efficient in front of proline residues. When proteins with many proline residues are of particular interest, consider the usage of other proteases.
    ***Note:*** Generally, a protein to trypsin ratio of 1:20 - 1:100 is optimal. 250 ng trypsin (0.5 μL from the stock solution) is recommendable for low but unknown protein concentrations.
    **CRITICAL:** Use sequencing-grade modified trypsin to ensure the specificity of trypsin for proteolysis at the carboxylic side of lysine and arginine. Native trypsin shows a broader specificity (chymotrypsin-like activity). Fragments from non-tryptic peptides can interfere with database searching from LC-MS data. Furthermore, native trypsin generates more trypsin autolysis products, which can interfere with and suppress sample-derived tryptic peptides in LC-MS measurements. If highly abundant trypsin autolysis peaks are visible in the LC-MS measurements, reduce the trypsin amount (problem 6).
  j. Perform tryptic digestion at 37°C between 4 and 16 h.
    **CRITICAL:** Do not perform tryptic digestion for shorter than 4 h or longer than 16 h. Short digestion times reduce digestion efficiency. Long digestion times lead to more trypsin autolysis products and unspecific cleavages that reduce the protein identification rate and quantification accuracy.
  k. Quench tryptic digestion by acidifying the digestion mixture to a final concentration of 1% (v/v) formic acid (FA).
  l. SDC precipitates at low pH values. Centrifuge at 20,000 × *g* to sediment precipitated SDC samples for 10 min.
  m. Transfer the supernatant to a new vial and dry in a vacuum centrifuge (SpeedVac SC110 Savant, Thermo Fisher Scientific, Bremen, Germany).
  n. The lyophilized peptides are stable between +20°C and 25°C and can be stored until further usage or shipped for external measurements without further cooling.^36^
    **CRITICAL:** The supernatant should be transferred carefully with a narrow pipette tip, and SDC should not enter the LC-MS system. For vacuum centrifugation, turn on the rotation speed before the vacuum pump.
9. Tandem Mass Tag (TMT) 10 plex labeling.
  In this step, label the tryptic peptides with ten different isobaric TMT labels. With TMT labeling, samples with different labels can be pooled and simultaneously analyzed. The signal intensity of peptides increases by up to 10-fold, boosting the protein identification rate for low sample amounts.
  ***Note:*** 1/10 downscaling was used for labeling low amounts of peptides.
  a. Dissolve each sample in 10 μL TEAB (100 mM) buffer. Vortex samples and incubate between +20°C and 25°C for 10 min.
  b. Solubilize dry TMT labels in 41 μL fresh LC-MS grade acetonitrile (ACN), vortex and incubate between +20°C and 25°C for 10 min.
  c. Add 4.1 μL of the assigned TMT reagent to each sample. Vortex the samples for 5 min, then label for 1 h between +20°C and 25°C at a rotation speed of 1,000 rpm in a ThermoMixC (Eppendorf, Hamburg, Germany).
  d. After 1 h, use 0.8 μL Hydroxylamine to quench the reaction for 15 min between +20°C and 25°C and a rotation speed of 1000 rpm in a ThermoMixC (Eppendorf, Hamburg, Germany).
  e. Immediately after labeling, combine equal amounts of each sample and dry it in a vacuum centrifuge (SpeedVac SC110 Savant; Thermo Fisher Scientific, Bremen, Germany). Store immediately at −80°C for further use.
    ***Note:*** Assign neighboring TMT labels for samples from the same group.
    ***Note:*** Here we used TMT 10 plex, allowing for the consecutive analysis of up to 10 samples at a time. When more than 10 samples are analyzed, multiple TMT batches must be measured.
    **CRITICAL:** After database searching, mathematically remove technical variances between TMT batches (batch effects). For this purpose, include an internal standard in each batch. Internal standards should consist of equal amounts of all samples analyzed in a study. Isobaric labels, assigned to internal standards, should be switched between TMT batches. As an alternative, use TMT 16 plex that enables the consecutive measurement of up to 16 samples.
    **CRITICAL:** Carefully note the assignment of TMT labels to sample IDs.
    **CRITICAL:** For TMT10/11 and 16 plex, a mass difference of 6.3 mDa is expected between neighboring reporter ions.^37^ Hence, a highly resolving mass spectrometer is required.
10. Offline basic Reversed Phase (RP) HPLC fractionation
  a. Fractionate multiplexed TMT-labeled peptides to 13 fractions; this step reduces the complexity of each measured LC-MS run and increases the protein identification rate for each sample.
  b. For fractionation, use a monolith column (ProSwift RP-4H, 1 mm 3 250 mm (Thermo Fisher Scientific on an Agilent 12000 series HPLC system (Agilent Technologies) coupled to a fraction collector or similar methods. The high pH gradient consists of buffer A (Equilibration buffer): 10 mM ammonium bicarbonate (pH = 8.5) and buffer B (Elution buffer): 10 mM ammonium bicarbonate in 90% acetonitrile.
  c. To prepare the HPLC system, purge solvent lines A and B of air according to the manufacturer’s instructions.
  d. Equilibrate the RP fractionation column with 100% (v/v) B and 50% (v/v) A and B, respectively for 30 min.
    **CRITICAL:** The pump pressure must be constant at a given A/B ratio. Curved lines in the pressure profile indicate air in the system, in this case repeat the flushing and check the connections.
  e. Inject 50 μL of buffer A, and run as a blank gradient, to precondition the column.
  f. Run Quality Control (QC) before and after each sample fractionation to evaluate the column performance. For that, inject 50 μg of Pierce HeLa Protein Digest Standard (Thermo Fisher Scientific, Bremen, Germany) dissolved in 60 μL buffer A into the system.
  g. Before injection, dissolve the sample in 60 μL buffer A and centrifuge for 5 min at 20,000 × *g* to pellet the insoluble material.
    **CRITICAL:** Dissolve the dried peptides in 60 μL buffer A and inject 50 μL of that to avoid air bubbles into the system during sample injection.
  h. Transfer the supernatant to an injection vial and inject 50 μL of the sample into the sample loop of the HPLC system. To separate TMT-labeled peptides, use the following gradient, separating peptides within a 25-min gradient, linearly increasing from 3%–35% buffer B: (Table 1). After peptide separation, wash the column with 98% buffer B for 10 min, followed by 5 min reconditioning step to 3% buffer B.
    **CRITICAL:** Extracted protein amounts can vary between cell types. As protein amounts extracted from cells after sorting by flow cytometry are usually too low for protein concentration estimation, higher or lower amounts may be present than expected. Inject 50 μg of labeled peptides for ideal performance. If the chromatogram significantly deviates from the peptide standard (increased system pressure, higher chromatographic intensities, especially in the flow trough), further dilute the sample to prevent overloading the column. Low to undetectable intensities indicate the presence of lower sample amounts than expected. Consider LC-MS measurements without previous fractionation to prevent additional sample loss on the chromatographic column.

**Table 1:** Timetable summarizing the gradient for the basic reversed-phase (RP) chromatography, used for the fractionation of TMT-labeled peptides

Time interval (min)	Gradient (percentage of buffer B)	Flow rate (nL/min)	
0	3	200	
5	3	200	
30	35	200	
30.1	98	200	
40	98	200	
40.1	3	200	
45	3	200
  i. Collect 32 1-min fractions at a flow rate of 200 nL/min and combine them into 13 fractions (Figure 8).
  j. Dry the 13 fractions in a vacuum centrifuge (SpeedVac SC110 Savant; Thermo Fisher Scientific, Bremen, Germany) and immediately store them at −80°C until further use.
    **CRITICAL:** After separation, a blank gradient should be run through the column, followed by water and methanol at a flow rate of 1 mL/min for at least 30 min. The column should not be stored in basic solvent as this can cause peak widening due to premature ageing of inappropriately stored columns.
11. LC-MS^3^ measurement.
  This step measures TMT-labeled peptide fractions using a low pH RP-LC-MS^3^ method at a nano-UPLC system (Dionex Ultimate 3000 Thermo Fisher Scientific, Bremen, Germany); Trapping column (for desalting and purification): Acclaim PepMap 100, 100 μm × 2 cm, 100 Å pore size, 5 μm particle size (Thermo Fisher Scientific, Bremen, Germany); Analytical column: Acclaim PepMap 100, 75 μm × 50 cm, 100 Å pore size, 2 μm particle size (Thermo Fisher Scientific, Bremen, Germany)) coupled to a Quadrupole-Orbitrap-Iontrap Tribrid Mass Spectrometer (Orbitrap Fusion, Thermo Fisher Scientific, Bremen, Germany).
  a. Buffer A (Equilibration buffer): 0.1% FA in LC-MS grade water (pH = 3.0) and buffer B (Elution buffer): 0.1% FA in ACN forms the low pH gradient of the UPLC.
    ***Note:*** 1.9 μg peptides can be expected in every fraction, as peptide yields are approximately half the original protein yields.
  b. Before LC-MS/MS analysis, solubilize the dried peptides for each fraction in 10 μL 0.1% FA (pH 3).
  c. Inject 50 μL of buffer A, and run as a blank gradient, to precondition the column.
  d. Run Quality Control (QC) before and after each set of 13 fractions to evaluate the column performance by injecting 1 μg of Pierce HeLa Protein Digest Standard (Thermo Fisher Scientific, Bremen, Germany) dissolved in buffer A to a final concentration of 1 mg/mL.
  e. After QC, inject 5 μL of buffer A into the system and run as a blank gradient to clean the column.
  f. Inject 5 μL (∼1 μL ) samples for each fraction to analyze TMT-labeled peptides. Select LC and MS parameters as described in Tables 2 and 3.
    ***Note:*** While measuring continuous fractions from one TMT 10-plex batch, there is no need to run blanks between fractions. While using multiple batches, run blank and QC samples between batches but not between fractions of one batch.
    ***Note:*** Use MS^2^ and MS^3^-based methods to identify and quantify TMT-labeled peptides. MS^3^ methods result in higher protein ID rates^38^ and a more accurate quantification for TMT-labeled peptides.
    **Pause point:** until downstream data analyses.

**Table 2:** Timetable summarizing the gradient for the low pH RP chromatography, used online before MS^3^ analysis of TMT labeled peptides

Time interval (min)	Gradient (percentage of buffer B)	Flow rate (nL/min)	
0	2	300	
10	2	300	
70	30	300	
70.1	90	300	
75	90	300	
75.1	2	300	
90	2	300

**Table 3:** Mass spectrometric (MS) parameters used for the LC-MS^3^ analysis of TMT-labeled peptides

Parameter	Value
**Polarity**	Positive
**Source**	ESI
**Spray Voltage**	1800 V
**Time between master scans (s)**	3
**Full MS**	
Detector type	Orbitrap
Orbitrap Resolution	120000
Automated gain control target (AGC Target)	2 × 10^5^
Maximum injection time (ms)	120
Scan Range (m/z)	380–1500
**dd-MS**^**2**^	
Detector type	Ion trap
Ion trap speed	Rapid
Automated gain control target (AGC Target)	1 × 10^4^
Maximum injection time	50 ms
Scan Range (m/z)	400–1200
Activation type	Collision induced dissociation (CID)
CID energy(%)	35
Monoisotopic peak determination	Peptide
Charge exclusion	Include 2-6
Dynamic precursor exclusion	30 s
**dd-MS**^**3**^	
Detector type	Orbitrap
Orbitrap Resolution	50000
Automated gain control target (AGC Target)	5 × 10^4^
Maximum injection time (ms)	86
Scan Range (m/z)	120–500
Activation type	Higher energy collision induced dissociation (CID)
HCD energy (%)	65
Precursor selection mode	Synchronous precursor selection (SPS) Top 10

### Bioinformatic data analyses

**Timing: >1 day**

Here, we describe examples of bioinformatic analyses applicable to both proteomic and transcriptomic data.

***Note:*** Consider that especially the proteomic data acquisition is subject to detection limits (see problem 7).

12. Analyses for “omics” data.
  a. For detailed parameter settings settings in proteomic data analysis, see Table 4. Search LC-MS^3^ against a mouse FASTA database using the SEQUEST algorithm integrated into Proteome Discoverer 2.4 (Thermo Scientific, Bremen, Germany).
    ***Alternatives:*** As an alternative to Proteome Discoverer, use freeware software for protein identification from LC-MS data, such as MaxQuant or the openMS platform, provided they are compatible with TMT 10-Plex data.
    **CRITICAL:** Search all 13 fractions from one TMT 10-Plex batch together as fractions.

**Table 4:** Parameters used for database-searching of LC-MS^3^ raw data of TMT-labeled peptides in Proteome Discoverer 2.4

Parameter	Value
**Modifications**	
Fixed modifications	Carbamidomethyl (Cysteine)
	TMT 10-Plex (Lysine, Peptide N-Terminus)
Dynamic modifications	Oxidation (Methionine)
	Acetyl (Protein N-Terminus)
	Deamidation (Protein N-Terminus)
	Methionine Loss (Protein-N-Terminus)
	Methionine Loss (Protein-N-Terminus) + Acetyl (Protein N-Terminus)
	PyroGlu (Glutamine)
**Digestion**	
Protease	Trypsin (Proteolysis before Praline enabled)
Missed cleavages	2 allowed
**Peptide properties**	
Maximum charge	6
Maximum amino acid number	6
Maximum amino acid number	144
Maximum weight (Da)	350
Minimum weight (Da)	5000
**Mass tolerance**	
Precursor mass tolerance (ppm)	10
Fragment mass tolerance (Da)	0.6
Reporter mass tolerance (ppm)	20
**FDR calculation**	
PSM FDR	< 0.01
Peptide FDR	< 0.01
Protein FDR	< 0.01
**Reporter quantification**	
Peptides to use	Unique and Razor
Co-Isolation threshold	50
Average reporter Signal to noise ratio	10
SPS mass matches (%)	65
  b. Use the intensities or the label-free quantification (LFQ) values obtained from MaxQuant or the openMS software as input for the analysis of differentially expressed proteins. Several options exist to calculate log_2_ ratios with *p*-values and adjusted *p*-values, including the basic statistical tools available in Microsoft Excel or more specific and comprehensive R-packages such as DEP (https://bioconductor.org/packages/release/bioc/vignettes/DEP/inst/doc/DEP.html, last accessed on 31.10.2023).
    **CRITICAL:** Once the basic differences are calculated, apply different stringencies for exploratory data analysis. For discovery purposes, less stringent cut-offs and non-adjusted p-values can be used but in principle, they should be followed by subsequent biological validations. If possible, start with stricter cut-offs on the Bonferroni adjusted p-values (p.adj.) and on the log_2_ fold change (FC) differences (e.g., log_2_ FC differences of either >1 or < −1, corresponding to a ± 100% change concerning the initial value and p.adj. <0.001). The higher stringency allows concentrating on the more reliable biological effects and simplifies downstream experiments. If a larger cohort of candidates is required for further exploration, relaxing the cut-offs allows for a more significant number of proteins in the following steps. Subsequent cross-validation approaches are likely required to confirm the actual biological importance of the identified candidates in the process under investigation.
  c. For an initial exploration of the data, concentrate on the significantly changed proteins in control vs. experimental groups, starting with all candidates with padj < 0.001.
  d. Summarize the candidates as a dendrogram using a dendrogram() function in R package dendextend.^22^ Perform hierarchical clustering using the hclust() function for each protein with the significant values.
  e. Calculate the correlation between samples using the cor() function from the rstatix R package to analyze replicate and sample distribution.^20^ Furthermore, to visualize the results, use a heatmap2() function from the ggplot package.^39^ These results provide a measure of the variability between replicates.
  f. Several tools are available for Gene Ontology (GO) analysis. To get an overview over the generated data set use non-redundant functional gene ontology databases (biological process, cellular component, molecular function). Keep all the advanced parameters as indicated in the presets.
  g. One of these analysis tools is Gene set enrichment analysis (GSEA). GSEA can be performed with the online tool WebGestalt,^26^ freely accessible at http://www.webgestalt.org/, last accessed on 31.10.2023). With WebGestalt, one can perform several types of analyses. For details, see the step-by-step suggested WebGestalt analysis below.
    i. Take the list of proteins passing the defined fold change criteria for the selection (e.g., with p.adj. < 0.05) and use WebGestalt.^26^
    ii. In the basic parameters section, select the organism of interest, set the method of interest as "Gene Set Enrichment Analysis (GSEA)", the functional database as "geneontology", and the function database name as "noRedundant" in the functional gene ontology databases (including the three branches "biological process", "cellular component" and “molecular function”).
    iii. In the "Gene List" section, select the ID type corresponding to the ID of the proteins and either upload or paste the protein list organized in two columns where the first column is the protein ID and the second column contains the fold change values.
    iv. In the Advanced parameters section, select the significance level as FDR with 0.05 and click "submit". Now, generate the analysis report, and click on the Results Summary to download the results.
    v. For GSEA using the R package WebGestaltR, install the WebGestaltR package by
      >install.packages(“WebGestaltR”).
    vi. Use the following snippet of code for performing the GSEA.
      ***Note:*** GSEA could also be performed using clusterProfiler,^24^ an R Bioconductor package, which requires an R version 4 or greater.
      > library(WebGestaltR)
      > WebGestaltR(enrichMethod = "GSEA",
      > organism = "name_of_organism",
      > enrichDatabase = "name_of_enrichment_database",
      > enrichDatabaseFile = NULL,
      > enrichDatabaseType = "ensembl_gene_id",
      > enrichDatabaseDescriptionFile = NULL,
      > interestGeneFile = "path_to_the_protein_list_with_foldchange",
      > interestGene = NULL,
      > interestGeneType = "ensembl_gene_id",
      > collapseMethod = "mean",
      > minNum = 1,
      > maxNum = 500,
      > sigMethod = "fdr",
      > fdrMethod = "BH",
      > fdrThr = 0.05,
      > topThr = 10,
      > reportNum = 20,
      > perNum = 1000,
      > gseaP = 1,
      > isOutput = TRUE,
      > outputDirectory = "path_to_output_directory",
      > projectName = "Project_Name",
      > dagColor = "continuous",
      > saveRawGseaResult = FALSE,
      > gseaPlotFormat = "png",
      > setCoverNum = 50,
      > networkConstructionMethod = NULL,
      > neighborNum = 10,
      > highlightType = "Seeds",
      > highlightSeedNum = 10,
      > nThreads = 1,
      > cache = NULL,
      > hostName = "http://www.webgestalt.org/")
    vii. In the code, change the organism of interest and check the list of organisms using
      >listOrganism(hostName="http://www.webgestalt.org/",cache=NULL)
    viii. Change the enrichDatabse, check the list of available databases using
      >listGeneSet(hostName=”http://www.webgestalt.org/",cache=NULL)
    ix. At this point, run the code, and the store results file in the output directory.
    x. For functional enrichment analysis: STRING^25^ (https://string-db.org/ , last accessed on 31.10.2023) allows for the prediction of functional associations between proteins^40^; for details, see the step-by-step STRING analysis in the following steps of this section.
    xi. Take the list of all proteins passing the defined fold change criteria for the selection (e.g., with padj < 0.05).
    xii. Go to String, click on the tab "Proteins with Values/Ranks", and paste/upload the protein list with fold change values.
    xiii. Select the organism and set the FDR stringency in the Advanced settings menu to "high".
    xiv. Click on "Search" and wait to generate the analysis report. Then, visualize the results (proteome network, functional enrichment including gene ontology, pathways, protein domains and features).
    xv. For RNA-seq analysis, start with the standard workflow of DESeq2 version 1.43.0 (https://bioconductor.org/packages/devel/bioc/vignettes/DESeq2/inst/doc/DESeq2.html, last accessed: 31.10.23).
13. Integrate the “omics” data.

This protocol is tailored to use several modalities (e.g., proteomics, transcriptomics, but also DNA methylation analyses) and allows comparisons across multiple levels of biological complexity. This is particularly important for studying developmental stages. To this end, the user can implement bioinformatics tools that integrate and compare these data, such as the R package mixOmics and the DIABLO framework (http://mixomics.org/mixdiablo/, last accessed: 31.10.23).

## Expected outcomes

### In utero electroporation

This protocol describes the analysis of cell populations isolated from the developing mouse cortex. A key step of this method is the fluorescent labeling of distinct cellular cohorts by *in utero* electroporation of neuronal progenitor cells at specific time points during development to enable flow cytometry-based isolation of distinct cell types. Here, progenitor cells were co-transfected with a fluorescent marker and specific shRNA expressing plasmids or controls.

Use a promoter active in the relevant developmental stage to obtain enough sorted cells for the proteomic analysis at a given time during e.g., cortical migration. Promoters suitable for labeling distinct cell cohorts during development are pCBFRE and pHes5 in neuro**e**pithelial cells (NECs), pGlast in apical radial glial cells (aRGCs), Tα1p in basal intermediate progenitor cells (bIPCs) and in early neurons, or pNeuroD in early postmitotic neurons.^15^^,^^16^

Otherwise, the number of transfected cells for sorting by flow cytometry will not be sufficient. At the developmental stage of E12.5 → E14.5, the activity of the pNeuroD promoter is still remarkably low, and the transition from progenitor cells into early immature postmitotic neurons is not yet advanced enough to allow efficient flow cytometry-based isolation of postmitotic neurons for proteomic analysis. This can be ameliorated by e.g., implementing protocols based on transposable-mediated gene expression switch, such as.^41^ However, simultaneous transfection of a pCAG-driven fluorophore (e.g., pCAG-tDimer or pCAG-Venus) results in sufficient numbers of tDimer positive cells accumulated in the cortical plate. Thus, to label early born postmitotic neurons that eventually reside in cortical layers IV/V, transfection of a pNeuroD-driven transgene is recommended starting at early E13.^42^

It is important to confirm the sorting outcome via e.g., transcriptome or proteome analyses. Check for the presence of marker genes or common stably translated proteins for the target cell type. Here, we describe the isolation of apical radial glial cells (Figure 5A) and early postmitotic neurons (Figure 6A). In addition to mass spectrometry, the cellular identity was verified by bulk transcriptome analysis of flow cytometry sorted cells (Figure 7), confirming progenitor identity of pGlast-dsRed2 positive aRGCs^43^^,^^44^ versus immature neuronal identity of pNeuroD-eGFP positive cells^45^^,^^46^^,^^47^^,^^48^^,^^49^^,^^50^ and revealing a gradual transition from an aRGC to neuronal identity as we have reported previously.^51^

The Volcano plots in Figure 7 display genes compared between pGlast (E14) versus pNeuroD (E18), with marker genes highlighted in boxes. The (-)log_10_ (p.adj. values, DESeq2::rlog transformation) was plotted against the respective log_2_ fold change (l2FC). Genes shown in red passed the p.adj. cut-off of <0.01 and the l2FC above 2.00. Sophisticated neuronal progenitor marker genes were pronounced in E14 samples, whereas neuronal genes were yet downregulated (Figures 7A and 7D). Furthermore, RNA-seq data displayed as heatmap revealed the downregulation of sophisticated markers for neuronal progenitors, such as *Neurog1*,^52^^,^^53^^,^^54^ *Sox2*^55^^,^^56^^,^^57^ and *Pax6*^58^^,^^59^^,^^60^^,^^61^ from E14 to E18, and the upregulation of neuronal-specific markers, such as *Satb2*,^62^^,^^63^^,^^64^ *Unc5d*,^65^^,^^66^ and *Grin2b*^67^^,^^68^ from E14 to E18 (Figures 7B and 7E). Thus, neuronal progenitor genes were highly effective in excluding mature cell identity in E14 pGlast-dsRed2 samples, whereas some neuronal marker genes were already established in the E14 samples, even if not at high levels.

Note, that with the marker proteins (pCAG-Venus and pCAG-tDimer), we also targeted mainly neural progenitor cells and neurons, respectively (Figures 7D and 7E). This explains similar gene expression in progenitor (pGlast-dsRed2 labeled) and early neuronal (pNeuroD-eGFP labeled) target population, respectively, compare to control populations (pCAG-Venus and pCAG-tDimer, respectively), yet the enrichment of specific marker genes in the sorted target populations indicates a successful sorting and sequencing procedure (Figures 7C and 7F).

Note also that so-called “marker” genes, often derived from scRNA-seq analyses, do not necessarily correlate with the target cell type in development.

Under the control of the developmentally active promoters, the fluorophores were often weakly expressed in the lower cortical layers where the progenitor cells or the immature neurons were located or were still migrating. To improve the visibility of the transfected site, we co-transfected the expression plasmid encoding the fluorophore under the control of a developmentally active promoter with another expression plasmid encoding a different fluorophore that is driven by the ubiquitously expressed promoter pCAG, e.g., pGlast-dsRed2/pCAG-Venus or pNeuroD-eGFP/pCAG-tDimer (Figures 5 and 6). Thus, co-transfection of a different fluorophore driven by the ubiquitous pCAG promoter improved the identification and excision of the cortical transfection site. Furthermore, pCAG-driven fluorophores label a control cell population that can be isolated as a reference to the target cell population (e.g., expressing pGlast- or pNeuroD-driven fluorophores, Figures 5A and 6A) for subsequent RNA sequencing and comparison with the distinctive target cell population. Another advantage is that by co-transfecting fluorophores, one can transfect shRNA expressing plasmids and the scrambled shRNA control in the same litter using a different combination of colors, e.g., shRNA against Cep120/pNeuroD-eGFP and pCAG-Cerulean versus shRNA control/pNeuroD-eGFP and pCAG-tDimer – thus, allowing to differentiate the control and shRNA conditions by pCAG-Cerulean and pCAG-tDimer expression respectively.

### Flow-cytometry-based sorting and isolation of cells from developing mouse cortex

Pool cells from more than two embryos from the same experimental condition and from the same mother to collect ∼10,000 fluorescence-positive cells per sample for downstream analyses. The number of sorted cells after the expression of fluorophores under the control of a ubiquitously expressed promoter (e.g., pCAG) from a single brain is almost always sufficient. In contrast, the number of isolated cells after expressing the fluorophore under the control of a developmentally active promoter, such as pGlast (Figure 5) or pNeuroD (Figure 6) from a single preparation is often insufficient for proteomic analysis.

***Optional:*** The cells can also be stained with either an apoptosis marker or a viability marker to distinguish between living and dead cells. An FSC plot with viability staining is then generated.

The consecutive gating steps lead to a population (singlets SSC, Figure 4B) that is free of cell debris, multiple cell clusters and dead cells and from which the distinct neuronal populations expressing pGlast-driven dsRed2 (Figure 5A) or pNeuroD-driven eGFP (Figure 6A) and driven by developmentally active promoters can be isolated.

***Note:*** An average of ∼ 5 × 10^6^ cells is usually applied to flow cytometry, however, the number of distinct neuronal cells derived is always limited. Here, 20,000 cells expressing pGlast-dsRed2 after E12.5 → E14.5 transfection (1.07% pGlast-dsRed2 positive and 1.73% pCAG-Venus positive cells, Figure 5B) and 20,000 cells expressing pNeuroD-eGFP after E14.5 → E18.5 transfection (2.41% pCAG-tDimer positive and 0.79% pNeuroD-eGFP positive cells, Figure 6B) were isolated.

### Proteomic analysis of sorted cells by flow cytometry

LC-MS/MS analysis quantifies several thousand proteins at once. The exact number of proteins identified depends heavily on the cell type used and the amount of protein extractable from each sample. In our case, 1,093 proteins were identified.

Test the performance of the LC-MS system by evaluating the injection of Pierce HeLa Protein Digest Standard (Thermo Fisher Scientific, Bremen, Germany) before and after sample measurement. The expected outcome using the described LC-MS system and configuration are >3,200 proteins from 1 μg HeLa Standard. For more information on increasing the protein identification rate from cells sorted by flow cytometry, see the troubleshooting section under problems 4 and 5.

Use multiple replicates per condition to ensure high statistical quality. As a rule of thumb, a Pearson correlation of more than 90% can be expected between biological replicates. Samples that do not meet this criterion should be considered statistical outliers.

## Limitations

This method has the well-known limitation that it does not allow a quantitative comparison of the less abundant proteins in the isolated cells in the different experimental groups, as LC-MS/MS usually does not detect them (so-called “dropout”). Include an additional step of immunoenrichment following sorting by flow cytometry for very low abundant proteins. However, the additional step would require substantially more starting material. As alternative, use antibodies to enrich specific proteins or protein complexes. However, this approach would be a candidate-specific solution. For additional options to increase the protein identification rate, see the discussion in problem 7.

Another known caveat is the uneven expression level of pCAG-driven fluorophores mainly used for the identification of the cortical transfection sites compared to the expression level of developmentally regulated pGlast- or pNeuroD-driven fluorophores. The ubiquitous expression of the fluorophore driven by the pCAG promoter is much higher than the expression of the fluorophore driven by developmentally active promoters, especially by pNeuroD-eGFP when compared to pCAG-tDimer.

NeuroD is a bHLH transcription factor and a direct transcriptional target of NGN2.^69^ NeuroD1 is expressed in early postmitotic neurons in the subventricular and intermediate zone, but not in Nestin+ radial glial progenitors in the VZ as labeled by the chicken β-actin promoter of pCAG.^47^ Importantly, the level of protein expression in early neurons driven by the NeuroD promoter is transient and significantly lower than using the chicken β-actin promoter.^15^^,^^47^ While expression driven by progenitor-specific promoters, such as pGlast, is considerably higher and more robust than pNeuroD-controlled expression, pCAG-driven expression of the marker protein is still significantly higher than the developmentally-regulated pGlast-driven expression.^16^ Thus, expression of fluorescent reporter genes directly driven by cell-type-specific promoters frequently results in low levels of expression making these constructs impractical and at least challenging for later applications, such as detection of live, labeled cells.^70^

This general weakness of the method must be addressed by a rigorous compensation and gating strategy for the single transfected pCAG-driven fluorophores as depicted in Figures 5B and 6B. Therefore, run new compensation controls for each experiment with new transfections.

Fluorescence compensation is required in multi-color experiments to subtract the spectral overlap between fluorophores (incl. fluorescent proteins (FPs)). Spectral overlap or spillover means that fluorescence emission from one dye/FP spills into the range of the bandpass filter used for detection of the other dye/FP. If not appropriately compensated, this spillover results in the detection of false positive cells. Consequently, the ‘spillover signal’ needs to be subtracted from the total fluorescence signal intensity, which is referred to as (mathematical) compensation. Compensate e.g., using the “Compensation wizard” of the BD FACS Diva Software (Figures 5C and 6C) and run new compensation controls for each experiment employing new transfections.

Gene transfer by *in utero* electroporation into developing cortical cells in mice is efficient and transgene expression can persist for months in numerous cerebral targets, presenting several spatial and temporal advantages over other methods to study brain development *in vivo*.^7^^,^^70^^,^^71^^,^^72^^,^^73^^,^^74^^,^^75^ However, efficient control of expression of transgenes after *in vivo* electroporation can be ameliorated using protocols based on a transposable-mediated gene expression switch, such as.^41^

***Note:*** Highly autofluorescent cell types in the samples may be detected as a false, very low positive signal (lower than the actual signal) even in the FMO/single- and non-transfected samples. Do not include these cells in your final gates (as shown in Figure 5B).

***Note:*** Transfection of plasmids for marker protein expression under control of the pCAG promoter can easily lead to the presence of hundreds of gene copies in a single cell depending on the origin of replication, a sequence within the vector at which replication is initiated. The pCAG vector contains a pUC origin that can produce up to 500–700 copies per cell.^76^ Also, other transductions are conceivable: Cre/loxP-mediated inducible expression vectors can be used in combination with a vector expressing a conditionally active form of Cre recombinase for temporal regulation,^77^^,^^78^^,^^79^ which is activated by 4-hydroxytamoxifen. To achieve low vector copy numbers, virus-mediated transductions are recommended, including gene transfer by lentiviruses,^80^ adenoviruses,^81^ adeno-associated viruses^82^ and Herpes simplex viruses.^83^ Since functional gene analysis often requires both overexpression and downregulation of multiple, potentially interacting genes in the same cell, a sophisticated multicolor panel of lentiviral vectors was developed using the Lego "building block principle^84^: A full spectrum of fluorescent and drug-selectable marker genes allows simultaneous analysis of multiple gene expression patterns.

## Troubleshooting

### Problem 1

Post-surgery abortions (Step *In utero* electroporation).

Surgery is not always successful. The mice abort or reach a humane endpoint.

### Potential solution

• Always use endotoxin-free and high-quality DNA preparations for *in utero* electroporation.
• Maintain aseptic conditions – proper handling of mice and good post-operative care is mandatory to prevent abortions.
• Avoid electroporation of embryos closest to either side of the vaginal ligament – this also helps to avoid miscarriages.

### Problem 2

Low transfection rates (Step *In utero* electroporation).

Sometimes, the transfected cells are not visible either under the stereo microscope and/or during FAC sorting.

### Potential solution

• Measure the DNA concentrations for the plasmid mixes accurately using a NanoDrop Device such as Thermo Scientific NanoDrop Spectrophotometer and use freshly prepared plasmid mixes for IUE.

***Note:*** High-quality DNA preparations should have a high concentration for *in utero* electroporation to achieve a final DNA concentration in the plasmid mix from ∼4 μg/μL.

• Ensure proper electroporator settings and correct connection and orientation of the electrodes during electroporation.
• Ensure the reporter plasmids used for co-transfection are expressed adequately in the targeted cell types at the desired development stage (e.g., embryonically, or postnatally) – see the expected outcomes.

### Problem 3

Low yield after FACS/sorting by flow cytometry (Step Flow cytometry-based isolation of cells from developing mouse cortices).

Sometimes, fluorescent cells are visible, both under the microscope as well as during FACS, but the outcome later does not match this observation.

### Potential solution

• Low transfection rates (see problem 2).
• Check if the electrode is well-connected and is not compromised in current flow.
• If the expression of the fluorescence marker is low under the control of the developmentally active promoter, co-transfect with a ubiquitously active promoter, e.g., pCAG or pCMV, to identify the transfection site more efficiently and to ensure the efficient dissection of the transfection site. Uncut tissue leads to more cells to be sorted and therefore results in more time for cells to potentially become apoptotic prior to sorting.
• If the plasmid is weak, pool cells from more brains to meet the desired cell numbers (> 10,000 cells) for downstream analysis.
• Clogging of the nozzle is a common problem during flow cytometry-based sorting and can lead to reduced yield.

### Problem 4

Clogging of the nozzle during flow cytometry (Step Flow cytometry-based isolation of cells from developing mouse cortices).

Young neurons tend to constantly extend their neurites in the single cell suspension, which can lead to clumping within the flow cell and disturbed flow.

### Potential solution

• Digest thoroughly and triturate the cortical tissue. Resuspend in an EDTA-containing buffer followed by passing the cell suspension through a 40 μm insert filter.
• Once the cell suspension is applied to the cytometer, the sheath fluid hydrodynamically focuses the cell suspension in the flow cell above the nozzle and passes the cell suspension through the nozzle. We implemented a 100 μm nozzle for dissociated cortical neurons (Figure 4A). The fluid stream enables the cells to pass the laser light one cell at a time (Figure 4A).
• Nozzle sizes typically vary between 70, 85, 100, and 130 μm and the nozzle size should be about 4–5 times larger than the size of the cells. Lymphocytes and suspension cells are usually run through a 70 μm nozzle, while larger cells, such as adherent cell lines and dissociated primary cells are sorted with a 100 μm nozzle. Dissociated cortical neurons however are rather small in diameter: Pyramidal neurons have reportedly a soma diameter of 10–20 μm^85^^,^^86^ while all processes, dendrites and the axon are collapsed and retracted during dissociation.

***Note:*** Weigh the advantage of a larger nozzle size against a decreased flow rate that results in a prolonged sorting time that can ultimately lead to apoptosis and a reduced quality of the isolated proteins. Dissociated neural cells are susceptible to cell damage during the dissociation step, as their processes can be truncated, especially if the animals are past the optimal embryonic ages that are recommended for the preparation of dissociated cortical cells from mice or rats.^87^ Since early neurons, which invade the developing cortical plate, are either multipolar with several very minor processes or transition to a bipolar state with a short leading extension and a short tailing process,^88^^,^^89^^,^^90^ they are less susceptible to cell damage during dissociation. In our lab, this protocol was successfully applied to isolate dissociated primary neurons for either flow cytometric analysis or for primary culture of the isolated cells indicating that cells are unharmed and not damaged during dissociation.^1^^,^^2^^,^^3^^,^^91^^,^^92^^,^^93^ On the other hand, it has proven difficult to isolate intact adult corticospinal neurons from mice at postnatal age – while maintaining cytoplasmic integrity for comprehensive mRNA sequence analysis – as their large projection axon was reportedly lost upon dissociation and neuron fragility was related to axon length.^85^ Furthermore, the dissociation of primary neurons can cause neuron death, which often interferes with transcriptome or epigenome analysis, e.g., ATAC-sequencing protocols.^94^^,^^95^ Nevertheless, this protocol has been successfully applied for differential quantitative mass spectrometric proteomic analysis of migrating cortical neurons. The application on progenitor cells revealed expression changes in proteins involved in axonal formation and elongation, microtubule dynamics and polarized growth; all biological processes that occur within the axonal extension of a migrating neuron.^1^ This indicates uninjured cellular and axonal integrity during dissociation and isolation of cells, where the processes tend to be retracted but not cut off. Furthermore, this protocol allows an unbiased proteomic analysis of proximal somatic proteins and distal axonal or dendritic proteins localized in the processes.

### Problem 5

Contamination of the target cell population with control cells expressing pCAG-driven fluorophores (Step Flow cytometry-based isolation of cells from developing mouse cortices).

For some experimental questions, the control cells should not overlap in cell identity with the target population.

### Potential solution

• Ameliorate your compensation strategy:
• When compensating for a single transfected fluorophore, e.g., pCAG-tDimer, strive for optimal compensation and assure that the eGFP median fluorescent intensities of tDimer negative and tDimer positive populations are equal. Furthermore, compensation controls (FMOs, single transfected cells) must ideally be as bright or brighter than the experimental sample, otherwise there is a risk of under compensation in the experimental sample. Compensation is a mathematical process. Apply an automatic compensation, e.g., calculated by BD FACS Diva software.
• In case of a population of pNeuroD-eGFP/pCAG-tDimer positive cells nearly overlapping with the scattering profile of single-transfected tDimer positive cells, define a manual gating strategy that ensures the exclusion of single tDimer positive cells, e.g., by shifting the gating for eGFP to values clearly ≥ 10^3^, even if a significant proportion of pNeuroD-eGFP/pCAG-tDimer positive cells with a low expression of eGFP will not be isolated.

### Problem 6

A smaller number of proteins were detected by LC-MS/MS (Step Quantitative LC-MS/MS or RNA-seq).

LC-MS/MS is a sensitive method that is subject to detection limits, thus requires a relatively high amount of input, unlike RNA-seq.

### Potential solution

• Ensure clean preparation of cells and lysates used for LC-MS/MS analysis; this prevents contaminant proteins such as keratin from masking the less-abundant proteins.
• Test the quality of your tryptic digestion. The digestion efficiency can be analyzed by different methods, including the detailed investigation of the protein-sequence coverage and the cleavage specificity^33^^,^^34^ as well as the exponentially modified protein abundance index (emAPI).^35^ Inspect spectra manually for autolysis products from trypsin. High peaks for trypsin autolysis products indicate an excessive trypsin to protein ratio. Reduce the trypsin amount to a trypsin:protein ratio between 1:20 and 1:100.
• Test for the TMT-labeling efficiency as described.^96^
• Test the quality of your LC-MS/MS system and run Quality Control (QC) before and after each set of 13 fractions to evaluate the column performance by injecting 1 μg of Pierce HeLa Protein Digest Standard (Thermo Fisher Scientific, Bremen, Germany) dissolved in buffer A to a final concentration of 1 mg/mL.

### Problem 7

A limited number of identified candidates upon bioinformatic analyses (Step Bioinformatic data analyses).

In proteomic data analyses, batch effects and normalization methods can reduce the amount of differential detected proteins/potential candidates.

### Potential solution

• Increase the number of cells from sorting and increase the number of biological replicates analyzed to reduce variability.
• Avoid using a TMT approach or increase fractionation to reduce the complexity of your sample for each fraction, if enough material is available. As a rule of thumb, inject 1 μg protein into the LC-MS system for each fraction to provide optimal peptide identifications.
• Increase protein coverage (see problem 6).
• Reduce the stringency of the initial statistical analysis, bearing in mind that these candidates may in any case require subsequent biological validation.

## Resource availability

### Lead contact

Further information and requests should be directed to Froylan Calderon de Anda (froylan.calderon@zmnh.uni-hamburg.de) as the lead contact.

### Technical contact

Information and technical requests for resources and reagents should be directed to and fulfilled by Tabitha Rücker (tabitha.m.ruecker@gmail.com) and Hartmut Schlüter (hschluet@uke.de).

### Materials availability

This study did not generate new unique reagents.

### Data and code availability

The lead contact will provide the data and the code generated for the omic data analysis upon request. The published article includes the rest of the datasets/code generated or analyzed during this study.
